# VlbZIP30 of grapevine functions in dehydration tolerance via the abscisic acid core signaling pathway

**DOI:** 10.1038/s41438-018-0054-x

**Published:** 2018-09-01

**Authors:** Mingxing Tu, Xianhang Wang, Yanxun Zhu, Dejun Wang, Xuechuan Zhang, Ye Cui, Yajuan Li, Min Gao, Zhi Li, Yuejin Wang, Xiping Wang

**Affiliations:** 1 State Key Laboratory of Crop Stress Biology in Arid Areas, College of Horticulture, Northwest A&F University, 712100 Yangling, Shaanxi China; 2 Key Laboratory of Horticultural Plant Biology and Germplasm Innovation in Northwest China, Ministry of Agriculture, Northwest A&F University, 712100 Yangling, Shaanxi China; 3 Biomarker Technologies Corporation, 101300 Beijing, China

## Abstract

Drought stress limits the growth and development of grapevines, thereby reducing productivity, but the mechanisms by which grapevines respond to drought stress remain largely uncharacterized. Here, we characterized a group A bZIP gene from “Kyoho” grapevine, *VlbZIP30*, which was shown to be induced by abscisic acid (ABA) and dehydration stress. Overexpression of *VlbZIP30* in transgenic *Arabidopsis thaliana* enhanced dehydration tolerance. Transcriptome analysis revealed that a major proportion of ABA-responsive and/or drought-responsive genes are transcriptionally regulated by *VlbZIP30* during ABA or mannitol treatment at the cotyledon greening stage. We identified an *A. thaliana* G-box motif (CACGTG) and a potential grapevine G-box motif (MCACGTGK) in the promoters of the 39 selected *A. thaliana* genes upregulated in the transgenic plants and in the 35 grapevine homologs, respectively. Subsequently, using two grapevine-related databases, we found that 74% (23/31) and 84% (21/25) of the detected grapevine genes were significantly upregulated by ABA and drought stress, respectively, suggesting that these genes are involved in ABA or dehydration stress and may be regulated by *VlbZIP30* in grapevine. We propose that *VlbZIP30* functions as a positive regulator of dehydration-responsive signaling in the ABA core signaling pathway.

## Crop genetics: drought tolerance gene found in grape

Researchers in China have identified a key gene involved in drought resistance in grapes. After exposing grape plants to drought, Xiping Wang’s team at Northwest A&F University observed increased expression of the *VlbZIP30* gene. *VlbZIP30* expression also increased in response to the plant hormone ABA, a known regulator of stress response. Further investigation demonstrated that *VlbZIP30* probably regulates ABA signaling, though its precise activity remains unclear. The researchers discovered a genetic sequence consistently present in the promoters of genes activated by *VlbZIP30*. They also identified over 30 genes regulated by *VlbZIP30*. While many of these genes are probably involved in drought tolerance, more work will be needed to clarify their functions. These findings may serve to guide efforts to breed or engineer grape varieties with improved drought tolerance.

## Introduction

Grapevines are among the world’s major fruit crops, and their fruits can be consumed fresh or dried, be processed into wines, spirits, and vinegar, or be transformed into pharmaceutical products that promote human health^1^. However, abiotic stress, such as drought, perturb the metabolism and growth of grapevines, leading to a loss of yield and reduced fruit quality^2^. Consequently, increasing the resistance of grapevines to drought stress is an important factor in ensuring yield stability.

Stress signaling in plants can be transduced by various signaling components, including second messengers, signal transduction factors, hormones such as abscisic acid (ABA), and transcription factors (TFs). Such signaling associated with drought has been shown to cause changes in physiological, morphological, and molecular processes, including the activation of many drought-related genes and the accumulation of a range of proteins, reflecting a drought stress response^3^.

ABA is considered to be a stress hormone and is associated with drought tolerance, as well as involved in various developmental processes, including seed germination and seedling growth^3,4^. In the context of a drought response it has been shown to mediate stomatal closure and to promote cuticular wax biosynthesis^5^. ABA-mediated drought tolerance involves complex signaling networks, the core components of which have been identified. Briefly, when ABA is present, it binds to the ABA receptors PYR/PYL/RCAR (PYRABACTIN RESISTANCE1/PYR1-like/REGULATORY COMPONENT OF ABA RECEPTOR1), which interact with the PP2C (PROTEIN PHOSPHATASE 2C) proteins, forming a complex and releasing the inhibitory effect of PP2Cs on SnRK2 (SUCROSE-NONFERMENTING1-RELATED PROTEIN KINASE2) protein kinases. The activated SnRK2s proteins subsequently phosphorylate different downstream TFs, such as *AREB1* (ABA-RESPONSE-ELEMENT BINDING1) and *ABI5* (ABA INSENSITIVE5), which regulate the expression of ABA-responsive genes^6,7^.

TFs are generally identified according to conserved sequences, known as the DNA-binding domains. One of the largest TF families in higher plants is the bZIP family, members of which are characterized by a basic region/leucine zipper domain^8^. Previous studies have shown that bZIP proteins function as regulators of signaling networks by specifically binding *cis*-elements containing a core ACGT, such as the ABA-responsive element (ABRE; PyACGTGGC), the G-box (CACGTG), and the C-box (GACGTC)^9,10^, in the promoters of their target genes, to either activate or repress their expression^11^.

A number of studies have shown that bZIP TFs are important regulators of drought stress signaling. The involvement of bZIPs (*ABF1*, *AREB1/ABF2*, *ABF3*, *AREB2/ABF4*) in the regulation of drought responses was first reported in the model plant *Arabidopsis thaliana*^12–14^. Following these studies, drought-related bZIP genes have been identified in a range of other species, including *OsABI5* in rice (*Oryza sativa*)^15^, *LIP19* in wheat (*Triticumaestivum)*^16^, *ABP9* in maize (*Zea mays*)^17^, and so on.

In order to improve the drought resistance of grapevines, researchers have focused their attention on the identification of drought-related TFs. Several, such as *WRKY11*, *ERF1/2/3*, and *NAC26*, have been identified and their overexpression in *A. thaliana* has been shown to enhance drought resistance^18–20^. However, to date, only a few grapevine bZIP TFs have been functionally characterized during a drought stress response^21–24^, and their regulatory mechanisms are not well understood.

Early studies indicated that the bZIP TFs (especially group A) play an important role in the ability of plants to resist abiotic stresses^25^. Hereafter, we identified 47 bZIP genes in the grape genome, and of these it was found that the expression profile of bZIP30 (a group A bZIP TF) was upregulated in response to drought conditions^23^, suggesting that it may be associated with tolerance to drought stress. In this current study, we cloned the *VlbZIP30*, from “Kyoho” grapevine and ectopically expressed it in *A. thaliana*. The results of physiological and transcriptomic analyses of the transgenic lines are presented and its putative function in dehydration-responsive signaling via the ABA signaling pathway in grapevine is discussed.

## Materials and methods

### Plant material and growth conditions

Two-year-old “Kyoho” grapevine (*Vitis labrusca* × *V. vinifera*) plants used in this study were grown in the grapevine germplasm resource orchard of the Northwest A&F University, Yangling, Shaanxi, China (34°20′N, 108°24′E). The “Kyoho” grapevine was a Chinese variety from number 24635 in the Vitis International Variety Catalog. *A. thaliana* ecotype Columbia (Col-0) plants used as both wild-type (WT) and for transgenic experiments were grown in a greenhouse at 21 °C under long-day conditions (16 h light/8 h dark).

### Dehydration stress and ABA treatment of grapevine leaves

For dehydration treatments, grapevine shoots with three well-developed leaves were detached and immediately placed on dry filter paper in an illumination incubator with 16 h fluorescent light (12,000 lux)/8 h dark photoperiod at 25 °C, with a relative humidity of 60–70%. For ABA treatments, leaves were sprayed with 100 μM ABA (MP Biomedicals, LLc) while the shoots were immersed in water about 8 cm, and the plants were then placed under the same ambient conditions as above. Leaves from the same position were collected from three independent replicates of each treatment at 1, 2, 4, 6, 9, 12, and 24 h after initiating treatment. The 0 h samples were collected before each treatment was initiated and used as control samples according to the method described previously^20^. All samples were immediately frozen in liquid nitrogen and stored at −80 °C until further analysis.

### Bioinformatic analysis

Full-length amino acid sequences of bZIPs from *A. thaliana* and grapevine were obtained from The Arabidopsis Information Resource (TAIR; http://www.arabidopsis.org/index.jsp) and EnsemblPlants (http://plants.ensembl.org/index.html), respectively. Multiple amino acid sequence alignments were generated using DNAMAN software (Version 5.2.2.0, LynnonBiosoft, USA) with default parameters, and a phylogenetic tree was constructed using the neighbor-joining method and MEGA software (version 5.05), with 1000 bootstrap replicates, as previously described^21^. The predicted phosphorylation sites (C1, C2, C3, and C4) and highly conserved bZIP domain were analyzed as previously described^12^.

### Transformation and characterization of transgenic plants

The plant transformation vectors 35S:*VlbZIP30* and Pro_*VlbZIP30*_:*GUS* (β-glucosidase, details of vector construction are supplied in Supplementary Method S1) were transformed into *A. thaliana* by the floral dip method using *Agrobacterium tumefaciens* (strain GV3101)^26^. T3 homozygous lines from three independent transgenic lines were analyzed.

### Histochemical GUS assay

An in situ GUS activity assay was performed as previously described^21^.

### Osmotic stress and ABA treatment of transgenic seedlings

WT and transgenic seeds were harvested at the same time. For seed germination and cotyledon greening analyses, approximately 100 seeds from WT and each 35S:*VlbZIP30* line (OE1, OE6, and OE23) were grown on Murashige–Skoog (MS) medium^27^ (Sigma) solidified with 0.7% agar containing 2% sucrose with or without mannitol (300 and 350 mM) or ABA (0.5 and 1 μM), at 21 °C with a 16 h light/8 h dark cycle. Germination and cotyledon greening rates were defined as the obvious emergence of the seedling radicle through the seed coat and green coloration of cotyledons, respectively. The seedlings were sampled after counting to measure the endogenous ABA concentration.

For the osmotic stress and ABA treatments, 7-day-old WT and transgenic seedlings were transferred from MS medium plates into MS agar medium, or MS agar medium supplemented with 300 or 350 mM mannitol, or MS agar medium supplemented with 50 or 100 μM ABA. The root lengths were measured 7 days after the transfer. The relative electrolyte leakage and malondialdehyde (MDA) content were measured as previously described^21^, as was ABA concentration^22^.

### Transcriptome analysis and identification of differentially expressed genes (DEGs)

Seeds from WT and transgenic lines were cultivated on MS agar medium, with or without stress treatment (0.5 μM ABA or 300 mM mannitol) for 7 days, and collected for RNA extraction. For each RNA purification biological replicate, 300 seedlings of WT or the OE lines from three MS agar plates were pooled to form a single sample. Three independent RNA samples were used for each experiment.

Total RNA was extracted using the E.Z.N.A. Plant RNA Kit (Omega Bio-Tek, USA, R6827-01), according to the manufacturer’s protocol (Invitrogen). RNA concentration and integrity were confirmed using a NanoDrop 2000 spectrophotometer (Thermo Fisher Scientific, Wilmington, DE, USA) and an Agilent 2100 Bioanalyzer (Agilent Technologies, CA, USA). The construction of RNA-Seq libraries and sequencing were performed by the Biomarker Biotechnology Corporation (Beijing, China). The libraries were generated using the NEBNext UltraTM RNA Library Prep Kit for Illumina (NEB, USA) following the manufacturer’s recommendations. Sequencing of the purified libraries was carried out using an Illumina HiseqXten platform (Illumina, NEB, USA) generating paired-end reads. The raw reads were cleaned by removing reads containing adapter sequences, reads containing poly-N, and low-quality reads. The cleaned reads from each sample were aligned to the *A. thaliana* reference genome from TAIR using the Tophat2 software^28^. Gene expression levels were determined by fragments per kilobase of transcript per million fragments mapped (FPKM), and the DEGs were identified using edgeR software^29^, with a threshold of false discovery rate (FDR) < 0.05 and absolute log2FC (fold change) > 1. All raw sequence data in this study have been submitted to the NCBI Short Read Archive under BioProject accession number PRJNA419694.

### Transcriptome data analysis

The Venn diagrams were made using the BMK Cloud platform (www.biocloud.net). Annotations for DEGs were retrieved from TAIR. Gene ontology (GO) enrichment analyses were performed for the functional categorization of DEGs based on the PageMan profiling tool^30^ and Arabidopsis Functional Modules Supporting Data^31^. The grapevine orthologs of the *A. thaliana* genes were identified using TBLASTX software^32^ with the highest score. Motif predictions were performed using the promoter region 1500 bp upstream of the start codons of the *A. thaliana* (AT) and grapevine (VIT) genes using DREME software (http://meme-suite.org/tools/dreme). The heat maps were constructed using HemI software^33^. To identify the predicted grapevine genes, two grapevine-related ABA^1^ and drought stress^34^ databases were downloaded from the National Center for Biotechnology Information (NCBI) under BioProject accession number PRJNA369777 and the Gene Expression Omnibus database under the number GSE57669.

### RNA extraction and quantitative real-time PCR (qRT-PCR)

Total RNA was extracted from the grapevine leaves after ABA and dehydration treatment using the E.Z.N.A._Plant RNA Kit (Omega Bio-Tek, USA, R6827-01) following the manufacturer’s instructions. The qRT-PCR was analyzed as previously described^21^. The expression levels of the grape *ACTIN1* (VIT_04s0044g00580) or *A. thaliana ACTIN2* (AT3G18780) genes were used as references. The specific primers for qRT-PCR are listed in Supplementary Table S1.

### Statistical analysis

Data analysis was performed using Microsoft Excel (Microsoft Corporation, USA). The data were plotted using Sigmaplot (v. 10.0, Systat Inc., CA, USA). One-way ANOVA followed by Fisher’s least significant difference (LSD) or Student’s *t*-test analysis were performed to assess significant differences using the SPSS statistics 17.0 software (IBM China Company Ltd., Beijing, China). All experiments were repeated three times as independent analyses.

## Results

### Identification of VlbZIP30, a group A bZIP TF from grapevine

**Fig. 1:**
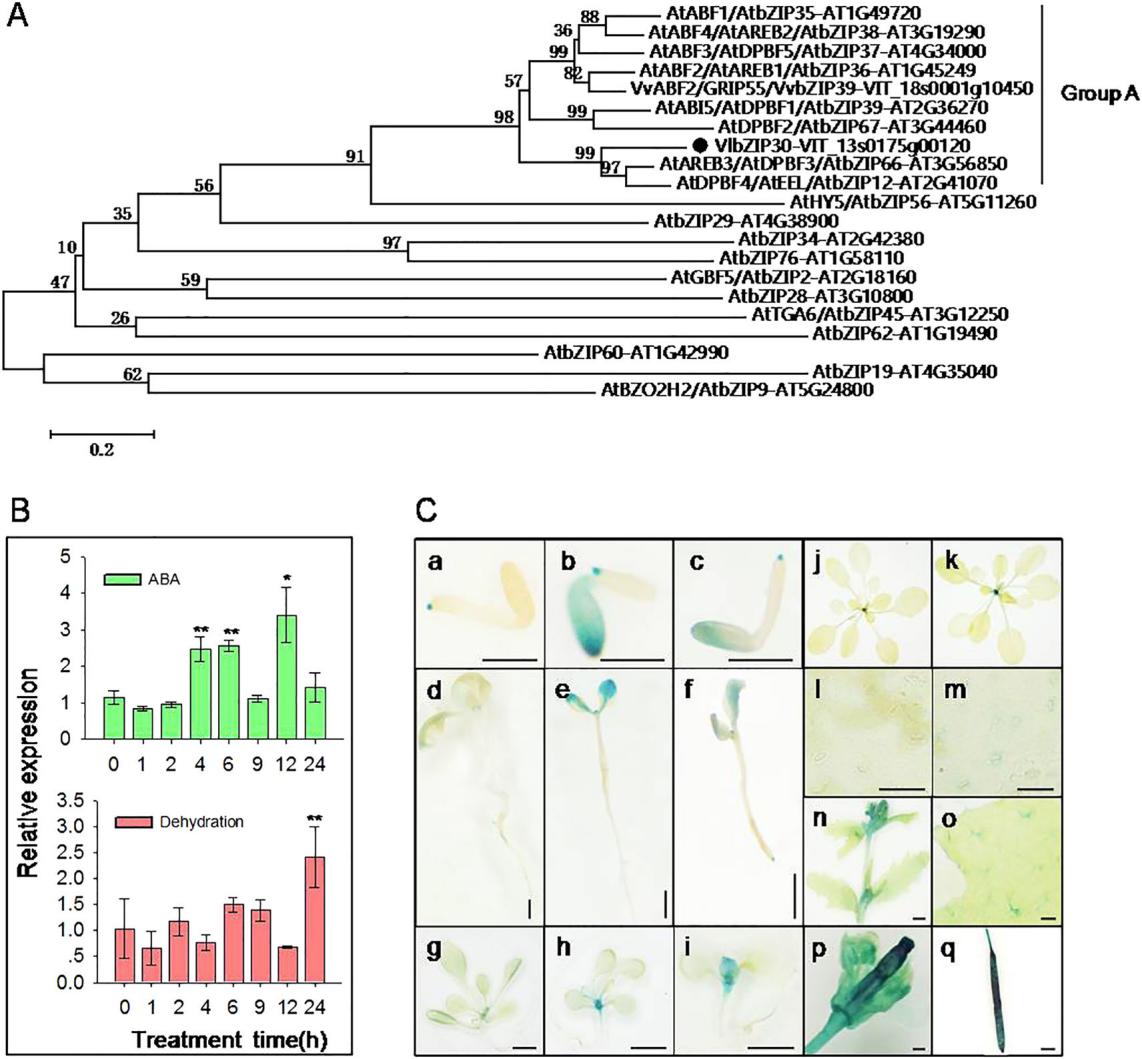
Phylogenetic analysis of VlbZIP30 and expression analysis of *VlbZIP30*. **a** The phylogenetic tree represents VlbZIP30 (black circle) and other bZIP amino acid sequences from *Arabidopsis thaliana* (AT) and grapevine (*Vitis vinifera*, VIT). The clustering of the group A bZIP proteins and the other groups of bZIP proteins (group H, AtHY5; I, AtbZIP29; E, AtbZIP34; L, AtbZIP76; S, AtGBF5; B, bZIP28; D, AtTGA6; J, AtbZIP62; K, AtbZIP60; F, AtbZIP19; and C, AtBZO2H2) have previously been reported^25,36^. **b** Expression profiles of *VlbZIP30* in grapevine following abscisic acid (ABA) and dehydration treatments. Data represent the mean values ± SE from three independent experiments. Asterisks indicate statistical significance (*0.01 < *P* < 0.05, **P < 0.01, Student’s *t*-test) between the treated and untreated control plants. **c** Patterns of *VlbZIP30* promoter-driven GUS (β-glucosidase) expression in *A. thaliana* at different growth stages. Mature embryos cultivated on Murashige–Skoog (MS) agar medium (a), or MS agar medium supplemented with 300 mM mannitol (b), or 0.5 μM ABA (c) for 2 days. Scale bar = 500 μm. Seven-day-old seedlings cultivated on MS agar medium (d), or MS agar medium supplemented with 300 mM mannitol (e), or 0.5 μM ABA (f) for 7 days. Scale bar = 500 μm. Fourteen-day-old seedlings transferred from MS medium plates into MS agar medium (g) or MS agar medium supplemented with 300 mM mannitol (h), or 100 μM ABA (i) for 7 days. Scale bar = 2 mm. 3-week-old plant (j). 3-week-old plant after dehydration for 2 h (k). Guard cells of 3-week-old plant (l). Scale bar = 50 μm. Guard cells of 3-week-old plant after dehydration for 2 h (m). Scale bar = 50 μm. Inflorescence (n). Scale bar = 2 mm. Leaf (o). Scale bar = 200 μm. Flower (p). Scale bar = 200 μm. Silique (q). Scale bar = 2 mm

The *VlbZIP30* (VIT_13s0175g00120) cDNA is 978 bp long and encodes a protein of 325 amino acids. Amino acid sequence analysis showed that, in common with the eight members of the *A. thaliana* ABF/DPBF bZIP subfamily, VlbZIP30 contains a basic leucine zipper domain^25^ and conserved domains predicted as phosphorylation sites (C1, C2, C3, and C4) involved in drought stress or ABA signaling^12^ (Supplementary Fig. S1). A phylogenetic analysis indicated that VlbZIP30 is most closely related to the group A ABF/DPBF TFs, which have previously been shown to be involved in ABA and drought stress signaling in *A. thaliana*^12–14,35^, and grapevine^22,36^ (Fig. 1a).

### Expression of *VlbZIP30* is induced by drought and ABA treatment

To test whether *VlbZIP30* is involved in ABA and drought stress signaling, we first evaluated the expression levels of *VlbZIP30* in grapevine following ABA or dehydration treatments using qRT-PCR. As shown in Fig. 1b, ABA caused an increase in *VlbZIP30* expression at 4 and 6 h shortly after initiation of the treatment. The expression peaked at 12 h, before decreasing for the next 24 h. Dehydration caused an increase in *VlbZIP30* expression at 24 h.

Next, to investigate the temporal and spatial expression patterns of *VlbZIP30* in more detail, histochemical GUS reporter experiments were performed with transgenic plants (Pro_*VlbZIP30*_:*GUS*) grown under ABA and dehydration stress, as well as under normal conditions. Low levels of GUS staining were observed in 2-day seeds at the germination stage, as well as in 7 and 14-day-old seedlings (Fig. 1c, a, d, and g), and GUS activity was significantly enhanced after mannitol (Fig. 1c, b, e, and h) and ABA (Fig. 1c, c, f, and i) treatments at the same stages. In mature plants, GUS staining was obviously detected in stems, trichomes, flowers, and siliques (Fig. 1c, n–q), while only slight staining was detected in leaf petioles (Fig. 1c, j), and no staining was detected in guard cells (Fig. 1c, l). However, after dehydration for 2 h, the leaf petioles and guard cells showed an increase in GUS staining (Fig. 1c, k and m). The dehydration treatments had no effect on the size of the stomatal aperture.

### Overexpressing *VlbZIP30* in *A. thaliana* reduces mannitol and ABA sensitivity during seed germination and post-germination growth

**Fig. 2:**
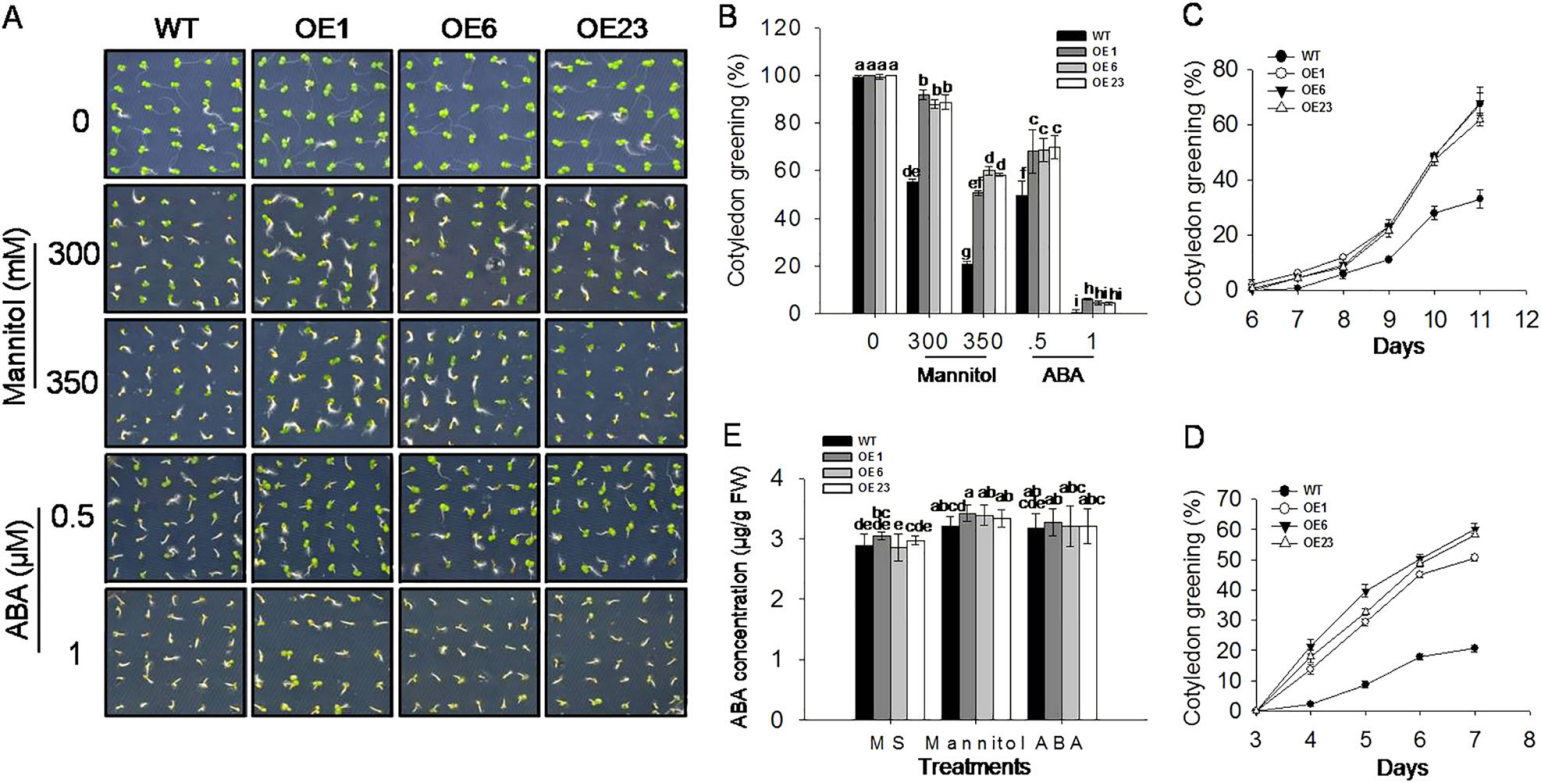
Phenotypes of wild-type (WT) and *VlbZIP30-*overexpressing (OE) transgenic lines at the greening cotyledon stage following mannitol and abscisic acid (ABA) treatments. **a** Greening cotyledons from WT and transgenic lines 7 days after seeds were cultivated on Murashige–Skoog (MS) agar medium, with or without 300 or 350 mM mannitol, or 0.5 or 1 μM ABA. **b** Cotyledon greening rates of WT and transgenic lines 7 days after cultivation on MS agar medium with or without 300 or 350 mM mannitol, or 0.5 or 1 μM ABA. **c**, **d** Cotyledon greening rates of WT and transgenic lines grown on MS basal medium containing 1 μM ABA (**c**) or 350 mM mannitol (**d**). **e** Endogenous ABA levels of WT and transgenic lines 7 days after cultivationon MS agar medium, or MS agar medium containing 350 mM mannitol or 1 μM ABA. Three independent experiments were performed with about 100 seeds per experiment. Data represent mean values ± SE from three independent experiments. Statistically significant differences are indicated by different lowercase letters according to Fisher’s LSD test (*P* < 0.05)

Three homozygous transformed lines (OE1, OE6, and OE23) with the highest levels of *VlbZIP30* expression were selected based on qRT-PCR analysis (Supplementary Fig. S2). Sterilized seeds of the transgenic lines and WT plants were cultivated on MS agar medium with or without mannitol (300 and 350 mM), or ABA (0.5 and 1 μM). The seed germination rates and the cotyledon greening rates of the transgenic lines were significantly higher than those of WT after 3 days (Supplementary Fig. S3A, B) and 7 days (Fig. 2a–d) under mannitol and ABA treatments, respectively. Given that ABA controls seed germination, and that its biosynthesis can be affected by abiotic stress^37,38^, we measured endogenous ABA concentrations. However, there was no significant difference in ABA concentrations between WT and transgenic lines under 350 mM mannitol and 1 μM ABA treatments at the seed germination stage (Supplementary Fig. S3C), as well as at the cotyledon greening stage (Fig. 2e).

**Fig. 3:**
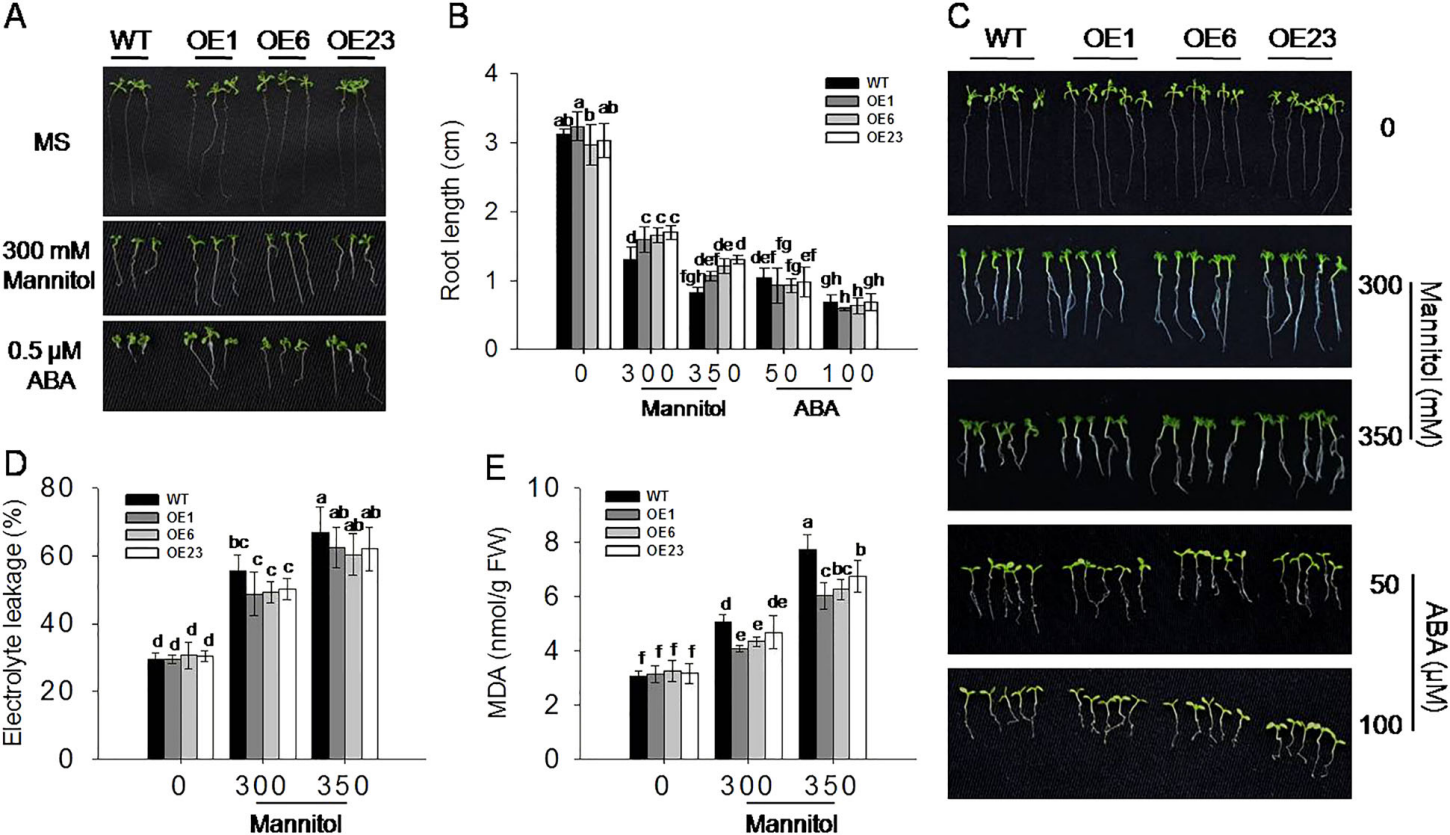
Phenotypes of wild-type (WT) and *VlbZIP30-*overexpressing (OE) transgenic lines at the post-germination growth stage during mannitol and abscisic acid (ABA) treatments. **a** Photographs of morphology in WT and transgenic lines 14 days after seeds were cultivated on Murashige–Skoog (MS) agar medium with or without 300 mM mannitol or 0.5 μM ABA. **b** Root length of WT and transgenic lines after 7 days of growth with or without mannitol (300 or 350 mM) or ABA (50 or 100 μM). **c** Photographs of 14-day-old seedlings transferred from MS agar medium to MS agar medium or MS agar medium supplemented with mannitol (300 or 350 mM) or ABA (50 or 100 μM) for 7 days. Electrolyte leakage (**d**) and malondialdehyde (MDA) content (**e**) of 14-day-old seedlings transferred from MS agar medium to MS agar medium or MS agar medium supplemented with mannitol (300 or 350 mM) or ABA (50 or 100 μM) for 7 days. In all cases, data represent mean values ± SE from three independent experiments. Statistically significant differences are indicated by different lowercase letters according to Fisher’s LSD test (*P* < 0.05)

To further characterize morphological changes of WT and transgenic seedlings in response to the mannitol and ABA treatments, the various seed genotypes described above were grown on MS agar medium, with or without 300 mM mannitol, or 0.5 μM ABA for 14 days. As shown in Fig. 3a, the size of cotyledons and roots of the transgenic seedlings were significantly bigger and longer than those of WT following both treatments, while there was no differences observed under control conditions.

To determine whether the transgenic lines had longer primary roots as a consequence of precocious germination or development of post-germination growth, 7-day-old transgenic lines and WT seedlings grown on MS agar medium were transferred to MS agar medium with or without mannitol or ABA, and grown for another 7 days. When grown on MS agar medium, the primary root lengths were similar; however, after treatments, the transgenic lines had relatively longer primary roots than WT seedlings under mannitol (300 and 350 mM) treatment, while there was no significant difference in response to the ABA (50 and 100 μM) treatment (Fig. 3b, c). This indicated that *VlbZIP30* plays a role in suppressing the retardation of germination mediated by ABA, but not in root growth inhibition.

Then, we measured electrolyte leakage and MDA levels in the transgenic and WT seedlings after 300 or 350 mM mannitol treatment and saw that electrolyte leakage levels between the transgenic lines and WT were no significant difference, but the MDA levels in the transgenic lines were significantly lower than in WT, indicating that the degree of membrane and tissue damage was less as a result of *VlbZIP30* overexpression (Fig. 3d, e).

### The expression of many ABA-responsive or drought-responsive genes is induced in *VlbZIP30*-overexpressing lines

**Fig. 4:**
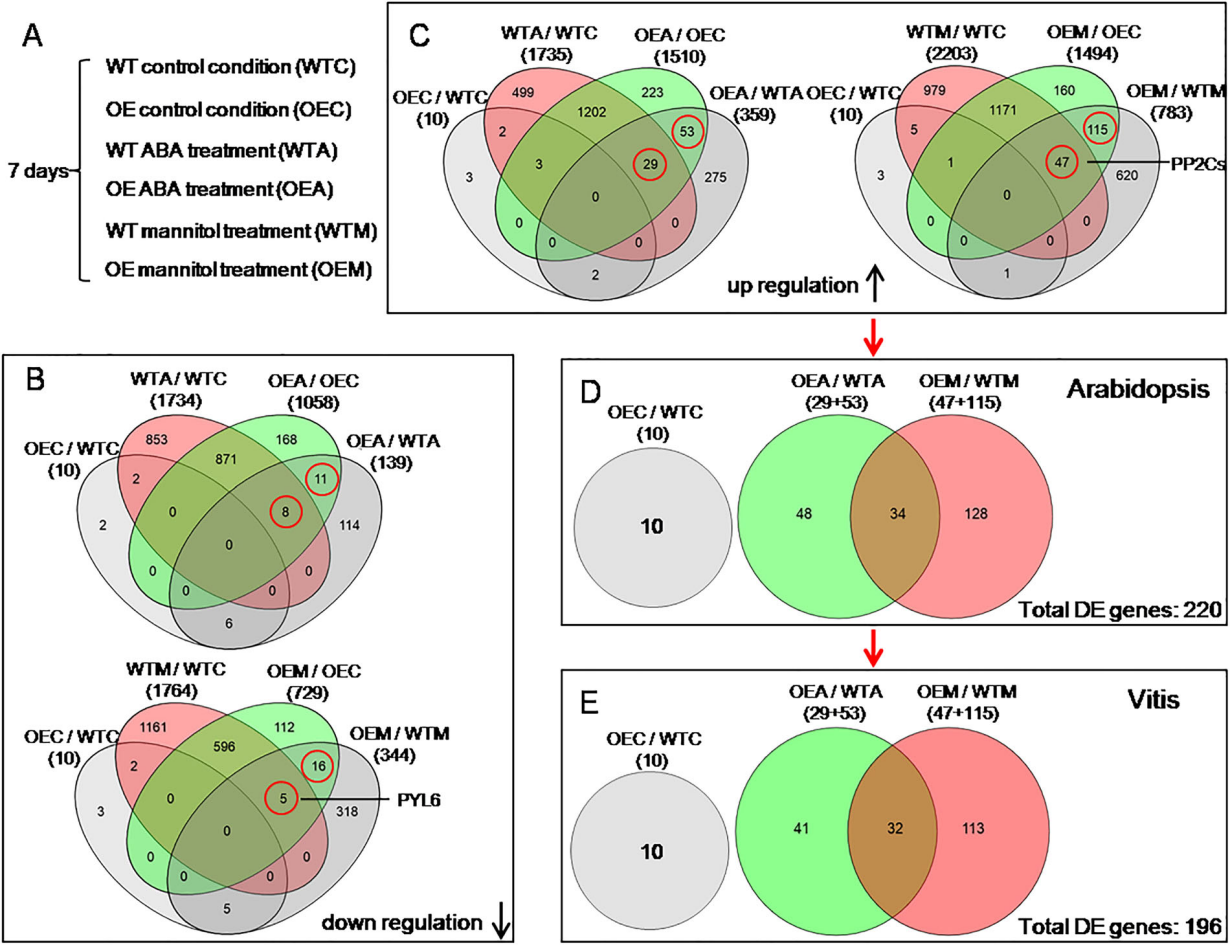
Venn diagram representation of the differentially expressed genes (DEGs) in four comparisons of wild-type (WT) and *VlbZIP30-*overexpressing plants (OE) grown under control conditions, abscisic acid (ABA) or mannitol stress. **a** Experimental set up: WT and OE transgenic seeds were cultivated on Murashige–Skoog (MS) agar medium (WTC, OEC) or MS agar medium supplemented with 0.5 μM ABA (WTA, OEA) or 300 mM mannitol (WTM, OEM) for 7 days. **b**, **c** show the numbers of overlapping downregulated and upregulated genes, respectively. The numbers in brackets represent the total numbers of DEGs in different comparisons. The DEGs in red circles were selected for further analysis. **d** The selected upregulated genes in the comparisons (OE/WT) under control conditions, ABA or mannitol stress in *Arabidopsis thaliana*. **e** The grapevine (*Vitis vinifera*) homologs of the selected *A. thaliana* DEGs from (**d**)

To examine the possible roles of *VlbZIP30* in transcriptional regulation in response to ABA and osmotic stress, we performed a global transcriptome analysis to identify DEGs between the WT and *VlbZIP30*-overexpressing lines using RNA-Seq. Seeds from WT and OE1 transgenic lines were cultivated on MS agar medium with or without 0.5 μM ABA or 300 mM mannitol for 7 days, and the seedlings were then collected for transcriptome analysis (Fig. 2a, scheme summarized in Fig. 4a). DEGs were defined based on a threshold of 2-fold change (FDR < 0.05). We identified 10 genes that were upregulated and 10 that were downregulated in the OE lines compared with WT plants under control conditions (OEC/WTC, Fig. 4b, c). Details of these 20 genes, including their annotation and their expression levels are listed in Supplementary Table S2. After treatments, a total of 1735 and 2203 upregulated genes and 1734 and 1764 downregulated genes were identified in WT plants subjected to ABA (WTA/WTC) and mannitol (WTM/WTC) stress, respectively (Fig. 4b, c). A total of 1510 and 1494 upregulated genes, and 1058 and 729 downregulated genes were found in the OE lines subjected to ABA (OEA/OEC) and mannitol (OEM/OEC) stress, respectively (Fig. 4b, c). We also identified 359 and 139 genes that were upregulated and downregulated, respectively, in the OE lines compared with WT plants when treated with ABA (OEA/WTA, Fig. 4b, c), while 783 and 344 genes were upregulated and downregulated, respectively, when treated with mannitol (OEM/WTM, Fig. 4b, c).

To deduce the possible functions of the *VlbZIP30*-induced genes, we performed a GO analysis of the genes, whose expression levels were significantly altered in the OE lines compared with the WT plants in response to ABA (OEA/WTA) or mannitol stress (OEM/WTM), using the PageMan profiling tool^30^. This revealed that some genes encode TFs, and some genes are putatively involved in photosynthesis, stress, signaling, transport, development, and several other metabolic pathways involving hormones, amino acids, and lipids (Supplementary Fig. S4).

To better understand the role of *VlbZIP30* in ABA and osmotic stress signaling, the OEA/OEC and OEA/WTA intersecting genes (upregulated, 29 + 53; downregulated, 8 + 11) and the OEM/OEC and OEM/WTM intersecting genes (upregulated, 47 + 115; downregulated, 5 + 16) were selected for further analysis (Fig. 4b, c). We identified all of the 248 (210 upregulated, 38 downregulated) genes mentioned above, and found that 38% (95/248) genes had been identified in *A. thaliana*. Among them, 54 genes were involved in abiotic stress, including stress-responsive genes (*RD20*, *RD22*, *RD26*, *SIS*, and *ERD10*), ABA signaling genes (*AFP1*, *AFP3*, *HB7*, *HB12*, *MAPKKK18*, *PYL6*), PP2C genes (*ABI1*, *ABI2*, *HAI1*, *HAI2*, *HAB1*, and *PP2CA*), TFs (*ABF2*, *ABF3*, *ABF4*, *DREB1A*, *NFYA5*, *NFYB2*, *NAP*, *MYB74*, *WRKY28*, *ERF053*, and *bHLH129*) (Table 1). The other 41 genes were involved in other processes such as wax biosynthesis, photosynthesis, transport, hormone signaling (brassinosteroids, ethylene, and cytokinins), and mineral homeostasis (Fe, Ca, phosphate, and sulfur) (Supplementary Table S3).

**Table 1:** Selected genes involved in abiotic stress with expression changes (FDR < 0.05) of at least two-fold in the *VlbZIP30* transgenic plants under different experiments from the microarray

To confirm the RNA-Seq results, we examined the expression of 20 drought-responsive genes by qRT-PCR and saw that the expression changes of all these genes were similar in the RNA-Seq (Table 1) and qRT-PCR data (Supplementary Fig. S5).

### Identification of a potential G-box motif in *VlbZIP30*-induced genes

**Fig. 5:**
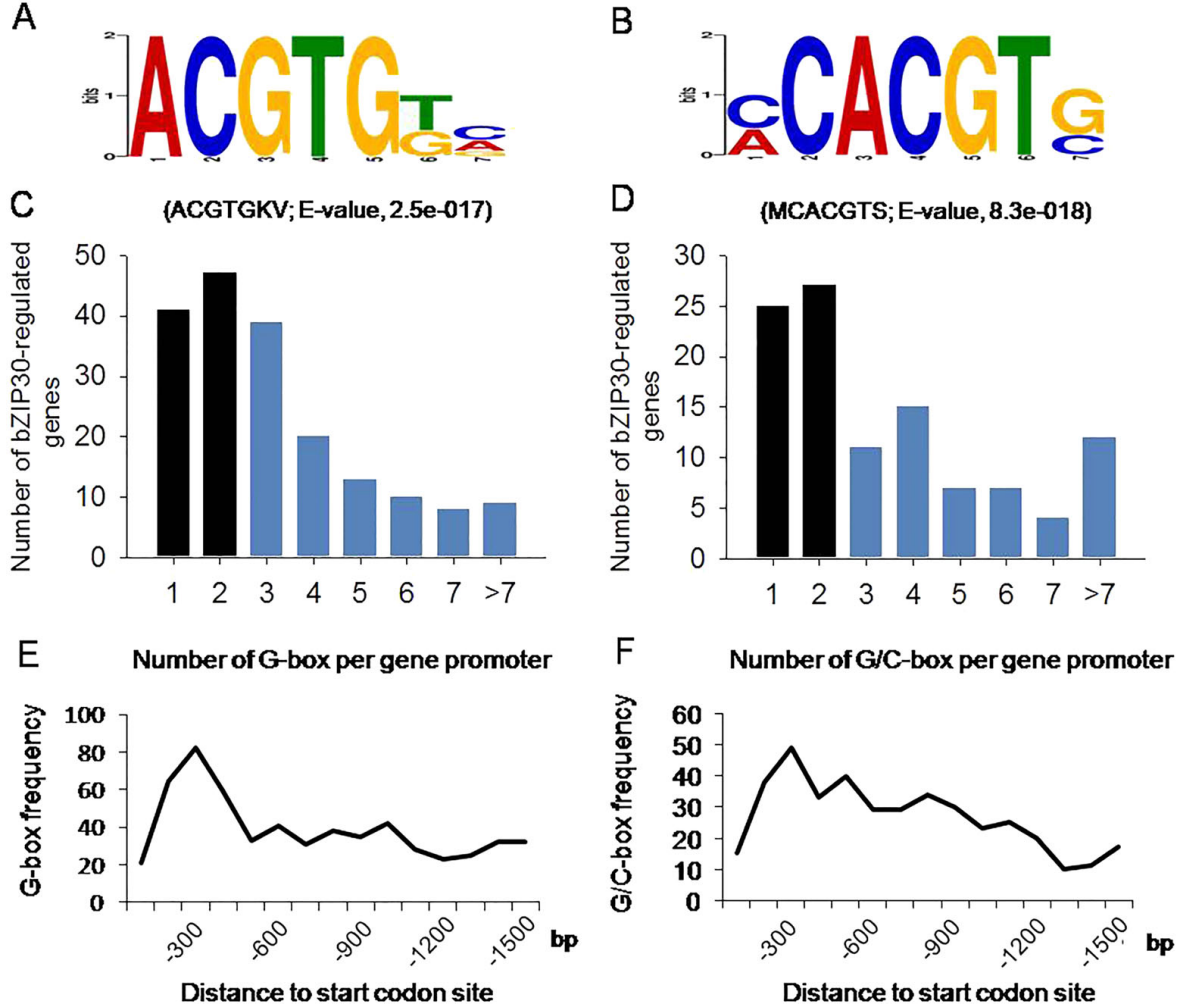
Enrichment of a potential G-box motif in *VlbZIP30*-induced genes. **a**, **b** DREME motif analysis, showing the predicted G-box (ACGTGKV) motif (**a**) in the promoter regions of selected *Arabidopsis thaliana* upregulated genes from the overexpressing (OE) lines, and the potential G/C-box (MCACGTS) motif (**b**) in the promoter regions of the corresponding grapevine (*Vitis vinifera*) homologs. **c**, **d** Number of predicted G-box motifs in the promoters of the selected *A. thaliana* genes (**c**), and the number of predicted G/C-box motifs in the promoter of the corresponding grapevine homologs (**d**). Predicted *VlbZIP30*-induced *A. thaliana* and grapevine genes with at least three G-box or G/C-box motifs were selected for further analyses (highlighted in blue). **e**, **f** Frequency of the predicted G-box motif in the promoters of the selected *A. thaliana* genes with respect to their distance from the start codon site (**e**), and the frequency of the potential G/C-box motif in the promoters of the corresponding grapevine homologs with respect to their distance from the start codon site (**f**)

To identify candidate *VlbZIP30* target genes, promoter analyses of the DEGs between OE lines and WT plants under control conditions (10 upregulated and 10 downregulated genes) and during ABA or mannitol treatment (210 upregulated and 38 downregulated genes) were performed using the DREME motif tool. We identified a potential G-box *cis*-element (ACGTGKV; E-value, 2.5e-017) including a 4-bp core sequence (ACGT) that is known to be a bZIP binding motif, as being significantly enriched in the promoters of the upregulated genes (Fig. 5a), of which 187 of the 220 genes carried this motif in their upstream 1500 bp promoter region. The G-box motif was not enriched in the downregulated genes. The number and location of the G-box motifs in the 187 gene promoters were analyzed, and many genes carried 1–3 G-box motifs, and that the highest G-box frequency was within the first 300 bp from the start codon site (Fig. 5c, e).

Then, we searched the grapevine genome for homologs of the identified *A. thaliana* upregulated (220) and downregulated (48) genes were identified (Fig. 4d, e; Supplementary Data S1) and a promoter analysis performed using the DREME motif discovery tool. Homologs were found of 195 of the upregulated and 44 of the downregulated genes (Supplementary Data S1). Using the same analytical method, surprising, a potential G/C-box *cis*-element motif (MCACGTS; E-value, 8.3e-018) including the core sequence (ACGT) was found to be significantly enriched in the homologs to the upregulated genes (Fig. 5b). We determined that 108 of the 195 genes had at least one G/C-box motif in their upstream 1500 bp promoter region, while this motif was not enriched in the downregulated genes. Many of the 195 genes carried 1 or 2 G/C-box motifs, and the highest frequency was within the 300 bp from start codon site (Fig. 5d, f), similar to the *A. thaliana* genes (Fig. 5c, e).

**Fig. 6:**
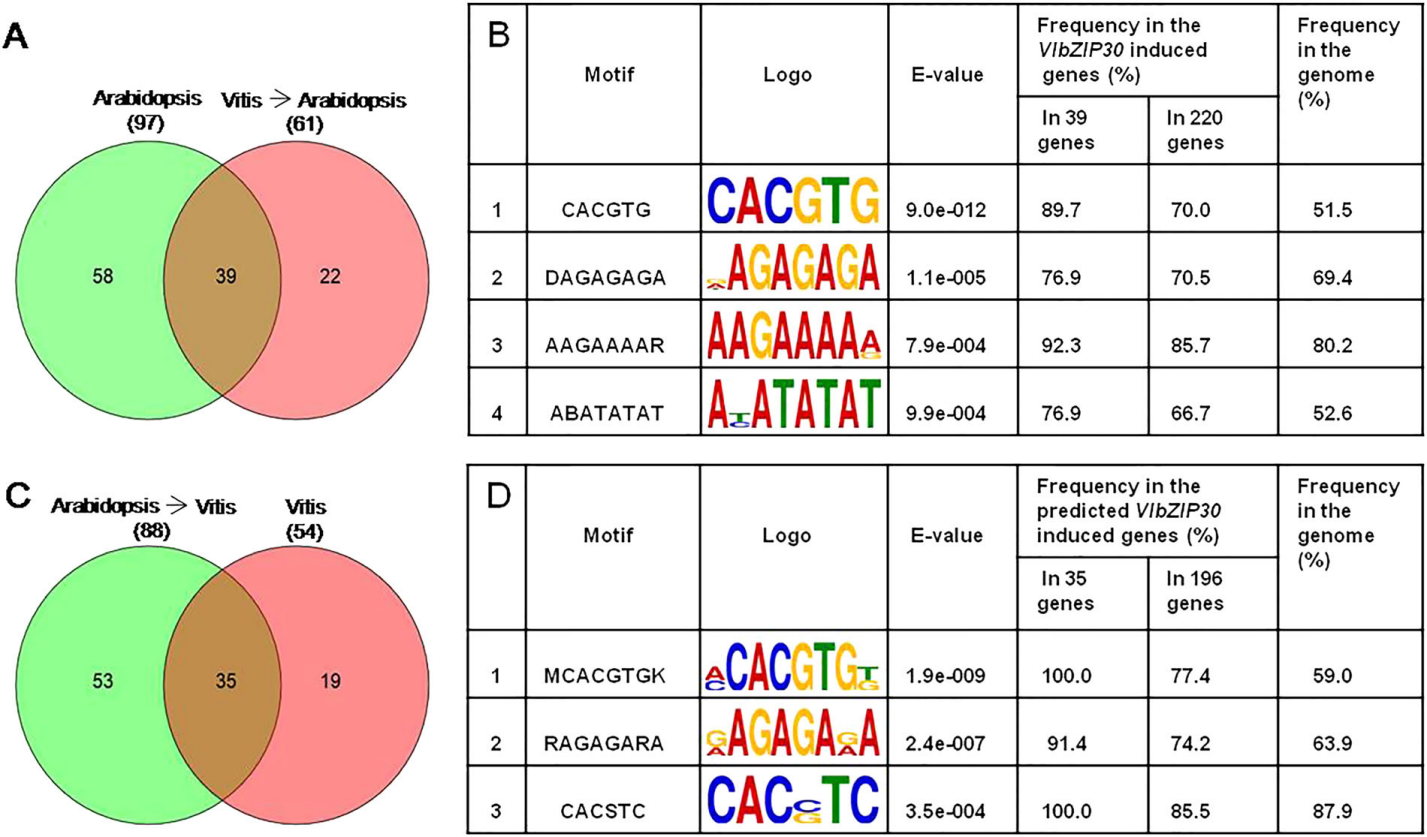
In silico promoter analyses of the selected *Arabidopsis thaliana* genes and the grapevine (*Vitis vinifera*) homologs. **a** Venn diagram showing the selected *A. thaliana* genes with three or more predicted G-box elements and the *A. thaliana* homologs of selected grapevine genes with three or more predicted G-box motifs. **c** Venn diagrams of the selected grapevine genes with three or more predicted G-box motifs and the grapevine homologs of the selected *A. thaliana* genes with three or more predicted G-box motifs. **b**, **d** The 1500 bp promoter regions of the overlapping 39 *A. thaliana* genes from (**a**) and 35 grapevine genes from (**c**) were analyzed using the DREME motif enrichment tool. The potential *A. thaliana* G-box and grapevine G-box motif enrichments were identified using the whole-genome sequences from *A. thaliana* and grapevine as a reference

Genes with three or more G-box (*A. thaliana*) or G/C-box (grapevine) motifs were selected for further investigation^39^. We found that 52% (97/187) of the *A. thaliana* genes and 50% (54/108) of the grapevine genes had at least three motifs and that all of the latter had G-box, but not C-box motifs. We found that 61 of the *A. thaliana* genes were homologous to 54 grapevines genes. Of the 97 and 61 *A. thaliana* genes, we identified 39 with three or more G-box motifs in their promoters shown as Venn diagram (Fig. 6a). Another DREME promoter analysis of these 39 genes revealed 4 enriched motifs, including a perfect G-box (CACGTG, E-value: 9.0e-012), a GAGA-box (DAGAGAGA, E-value: 1.1e-005), an AAGAAAAR motif (E-value: 7.9e-004), and a TATA-box (ABATATAT, E-value: 9.9e-004) (Fig. 6b). The frequencies of the G-box in the *VlbZIP30*-induced 39 and 220 *A. thaliana* genes were 89.7 and 70.0%, respectively, while the frequency in the whole genome was 51.5%, suggesting an enrichment in the *VlbZIP30*-induced genes. Such an enrichment was not found for the other three motifs (Fig. 6b).

In grapevine, 88 homologs of the 97 *A. thaliana* genes were found. Among the 88 and 54 grapevine genes, we identified 35 with at least three G-box motifs in their promoters shown as Venn diagram (Fig. 6c). Three enriched motifs, including a potential grapevine G-box (MCACGTGK, E-value: 1.9e-009), a GAGA-box (RAGAGARA, E-value: 2.4e-007), and a CACSTC motif (E-value: 3.5e-004) were identified in these 35 genes (Fig. 6d). Among them, the frequencies of the G-box in the predicted *VlbZIP30*-induced 35 and 196 grapevine genes were 100.0 and 77.4%, respectively, while the frequency of the G-box in the whole genome was 59.0%, again indicating an enrichment in the predicted *VlbZIP30*-induced genes. These results suggest that the 35 grapevine genes may be regulated by *VlbZIP30* via the potential G-box. The names and gene IDs of the 35 grapevine genes and 39 *A. thaliana* genes are listed in Supplementary Data S2.

### The expression of the predicted *VlbZIP30-*induced genes with at least three potential G-box motifs was upregulated in grapevine following both ABA and drought treatments

**Fig. 7:**
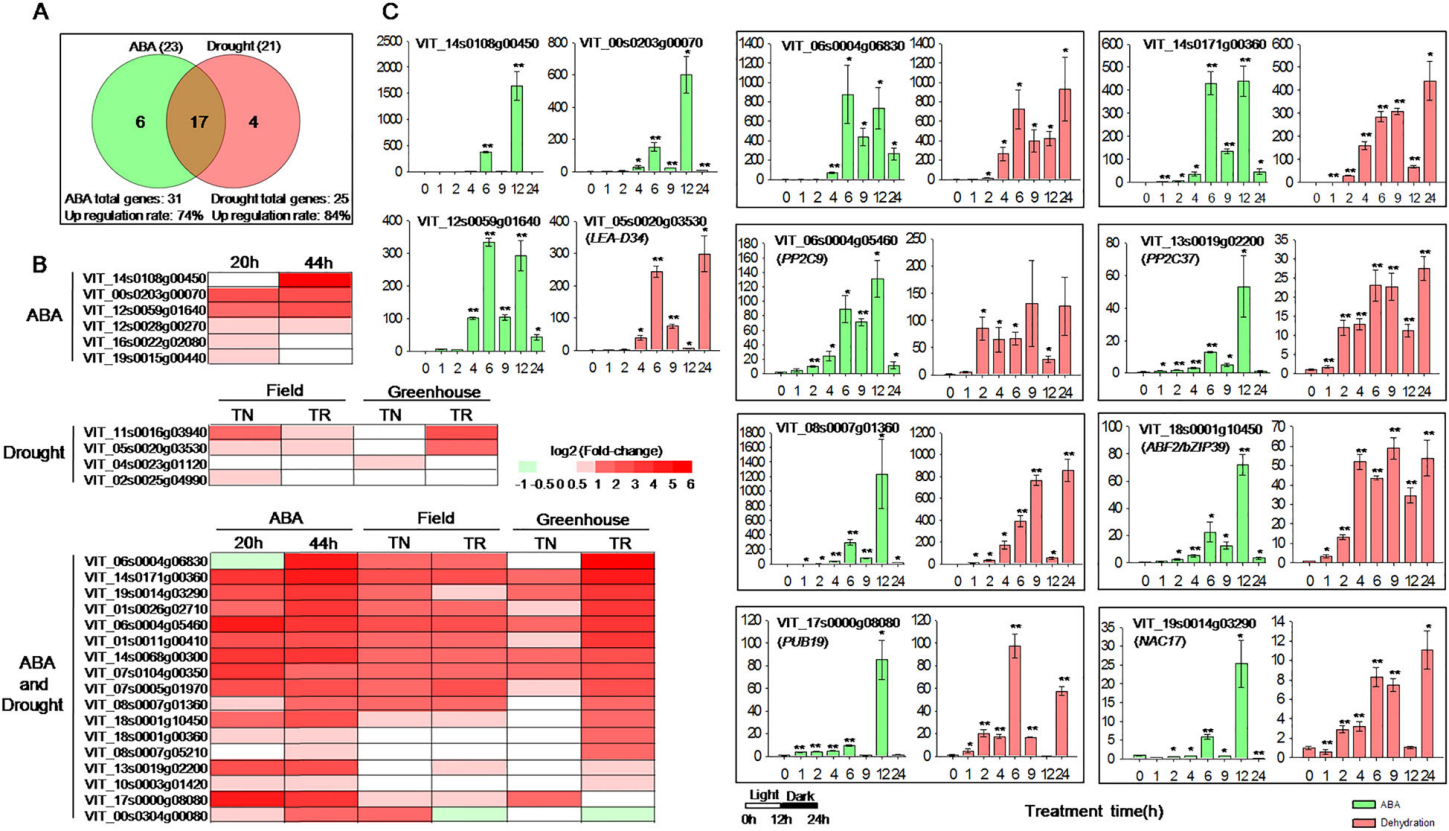
The expression profiles of predicted *VlbZIP30*-induced genes in grapevine (*Vitis vinifera*) following abscisic acid (ABA) or drought treatment. **a** Venn diagram of the selected upregulated genes in two different grapevine-related databases with the 35 predicted grapevine genes. Thirty-one and 25 predicted grapevine genes were present in the RNA-Seq and GeneChips^®^ data. The numbers in brackets represent the numbers of upregulated genes in the two different grapevine-related databases. **b** Heat maps showing the expression of the 27 genes found in (**a**). RNA-Seq analysis was performed of transcripts expressed in the skin of berries from grapevines treated, or not, with ABA for 20 and 44 h. The GrapeGene GeneChips^®^ data was derived from an analysis of the leaves of two *V. vinifera* L. varieties (Trincadeira, TR, and Touriga Nacional, TN) grown under control and drought conditions in a greenhouse, as well as under fully irrigated and non-irrigated conditions in the field. Heat map color gradation in red indicates the increase in expression (log2 fold change). **c** Gene expression profiles of randomly selected *VlbZIP30*-induced grapevine candidate genes analyzed using qRT-PCR. For each gene, the expression level in the 0 h sample from the ABA and dehydration treatments was defined as 1.0. The *VvActin1* gene was used as an internal control. Data represent mean values ± SE from three independent experiments. Asterisks indicate statistical significance (*0.01 < *P* < 0.05, **P < 0.01, Student’s *t*-test) between the treated and untreated control plants

To investigate the potential roles of the 35 identified grapevine genes in ABA and drought stress, two different grapevine-related databases for ABA^1^ and drought stress^34^ treatments were analyzed. RNA-Seq analysis was performed of grapevine berry skins with or without ABA treatment for 20 and 44 h. A GrapeGene GeneChips^®^ data analysis was performed on leaves from two *Vitis vinifera* L. varieties (Trincadeira, TR, and Touriga Nacional, TN) grown under control and drought greenhouse conditions, as well as fully irrigated and non-irrigated field conditions. We compared the genes in those datasets with the 35 predicted grapevine genes, and found that 31 and 25 appeared in the RNA-Seq and GeneChips^®^ data (Fig. 7a). Of these, 74% (23/31) and 84% (21/25) were significantly upregulated following the ABA and drought treatments, respectively (Fig. 7a). Six genes uniquely responded to ABA and 4 specifically responded to drought, while 17 genes responded to both treatments (Fig. 7a). The detailed expression data for these 27 genes (Fig. 7a) are shown in heat map diagrams in Fig. 7b.

We randomly selected 16 genes of the 27 genes induced by ABA or drought stress, for confirmatory qRT-PCR expression analysis, using the previously mentioned grapevine leaf samples subjected to ABA or dehydration treatment. The results were consistent with the results previously described (Fig. 7c; Supplementary Fig. S6).

## Discussion

Early studies identified three bZIP-type ABRE-binding proteins, *AREB1*/*2*/*3* from *A. thaliana*, using an ABRE motif^40^, and *AREB1*, *AREB2*, and *ABF3* were shown to play important roles in response to ABA and drought stress signaling^41^. In addition, Furihata et al. showed that exogenous ABA activates the SnRK2 protein kinases, and that the activated SnRK2 proteins phosphorylate a Ser/Thr residue in the conserved domains (C1, C2, C3, and C4) of the downstream *AREB1* gene, allowing it to bind to the *cis*-acting ABRE element of downstream drought-related genes^42^. The mechanism involving *AREB1* in the ABA core signaling pathway in *A. thaliana* is also present in economically important crops, such as rice^43–45^. These studies suggest that similar regulatory responses to stress are evolutionarily conserved in plants. In this study, we identified a group A bZIP TF, *VlbZIP30*, from grapevine. The results suggest that is positively regulates plant dehydration tolerance through regulation of the downstream drought-related genes via G-box *cis*-element (MCACGTGK) in ABA signaling pathway.

Previous studies have demonstrated that overexpressing drought-induced genes in *A. thaliana* can cause hypersensitivity to ABA and increase tolerance to drought stress^12^. However, in this study, overexpressing *VlbZIP30* in *A. thaliana* did not make the plants more sensitive to ABA, but increased their osmotic stress tolerance during germination and post-germination growth. Given that *VlbZIP30* is a TF, its role in osmotic stress response is likely to involve regulating downstream gene expression. Indeed, a majority of the ABA-induced and drought-induced genes tested were induced in *VlbZIP30*-overexpressing *A. thaliana* following ABA and mannitol treatment. Ninety-five (38%) of the 248 genes identified (Fig. 4) have previously been identified and of these 54 were involved in abiotic stress, including stress marker genes (*RD20*, *RD22*, *RD26*), ABA core signaling components (6 PP2Cs: *ABI1*, *ABI2*, *HAI1*, *HAI2*, *HAB1*, *PP2CA*, and *PYL6*), TFs (*ABF2*, *ABF3*, *ABF4*) (Table 1). Nine *A. thaliana* PP2Cs belonging to cluster A have been identified^46^, and studies have shown that six of them (*ABI1*/*2*, *HAI1*/*2*, *HAB1*, and *PP2CA*) function as negative regulators of ABA signaling, with their mutants showing hypersensitivity to ABA during seed germination and seedling growth^6,47–49^. In addition, the *ABI1*/*2* and *HAI1*/*2* genes are known to act in a negative feedback regulatory loop of the ABA signaling pathway^47,49^. A sextuple mutant impaired in six PYR/PYL receptors was shown to be very insensitive to ABA during seed germination and seedling growth^50^. Here, we found that the transcript levels of the six PP2Cs were all significantly higher in the *VlbZIP30-*overexpressing lines in response to osmotic stress, while the expression of *PYL6* was lower (Table 1). Consistent with this, the OE lines were found to be insensitive to ABA. These results suggested that *VlbZIP30* may play a role in a negative feedback regulatory loop of the ABA core signaling pathway under osmotic stress conditions in *A. thaliana*.

The ABRE (PyACGTGGC) and G-box (CACGTG) elements were identified as bZIP TF *cis*-binding elements regulating gene expression in response to ABA or drought stress in many plants, including *A. thaliana*^40^, rice^51^, wheat^52^, apple^53^, and so on. In this study, we identified 39 *A. thaliana* genes and 35 predicted grapevine genes (Supplementary Data S2) that may be directly or indirectly regulated by *VlbZIP30*. Seventeen (43.6%) of the 39 *A. thaliana* genes have been found to be involved in drought stress, including *RD26*, *AFP1/3*, *PP2CA*, *HAI1/2*, *ABF3*, *NAP*, *MYB74*, *WRKY28*, and *PUB19* (Table 1), implying that our analytical methods and results are credible. Other genes found here that have not been previously characterized may therefore also be involved in drought stress signaling. In contrast, there has been little characterization of the 35 grapevine genes, and only 3 were identified as being involved in ABA or drought stress signaling. *ABF2/bZIP39* (VIT_18s0001g10450), which was characterized as being involved in ABA signaling in grapevine cell culture, has been reported to transiently trans-activate the expression of *NAC17* (VIT_19s0014g03290) and *PUB19* (VIT_17s0000g08080) following ABA treatment^1,36^. In addition, overexpression of this gene in *A. thaliana* enhances tolerance to drought stress through the ABA signaling pathway^22^, suggesting that *NAC17* and *PUB19* may enhance drought stress in grapevine.

Subsequently, a perfect *A. thaliana* G-box (CACGTG) and a putative grapevine G-box (MCACGTGK) element were significantly enriched in the promoter of the 39 *A. thaliana* genes and 35 predicted grapevine genes (Fig. 6b, d). The highly conserved G-box motif (CACGTG) is regulated by bZIP TFs in organisms ranging from yeast to humans^54^. Ezer et al. constructed an available gene expression network (www.araboxcis.org) for prediction of genes regulating the G-box, or a set of genes regulated by the G-box. They identified approximately 2000 seedling-expressed genes expressed in 229 RNA-Seq samples of 7-day-old *A. thaliana* seedlings that are highly likely to be regulated by a perfect G-box motif (CACGTG) in their promoter, and predicted how bZIP proteins might regulate these genes^54^. These results suggest that *VlbZIP30* is likely to enhance *A. thaliana* dehydration tolerance by regulating downstream genes containing a perfect G-box (CACGTG). Large-scale transcriptome analyses also show that the G-box (CACGTG) was highly enriched in stress-responsive genes in grapevines^55^. These results suggest a general conservation in promoter framework, gene expression dynamics, and gene regulatory networks across species. We also used two grapevine-related databases to gain support for the potential roles of the 35 grapevine genes in ABA and drought stress.

We noted that 74 and 84% (a total of 27) candidate genes were significantly upregulated under ABA or drought treatment, respectively (Fig. 7a, b), and that the expression of which was up to 64-fold induced (Fig. 7b). In addition, the expression levels of 16 randomly selected genes from the 27 genes (including *VvPP2C9*, *VvPP2C37*, and *VvABF2*) were significantly upregulated by ABA or dehydration treatment by qRT-PCR analysis (Fig. 7c; Supplementary Fig. S6), supporting the accuracy of the RNA-Seq data. These results suggest that *VlbZIP30* may be involved in drought stress signaling in grapevine via regulation of the 27 grapevine genes containing the grapevine G-box (MCACGTGK). This conclusion is supported by the observation that 17 of the 39 *A. thaliana* homologous genes have previously been found to be involved in drought stress. Further studies be required to elucidate the functions of these regulated ABA and drought stress regulated grapevine genes.

## Electronic supplementary material

Supplementary Figure S1

Supplementary Figure S2

Supplementary Figure S3

Supplementary Figure S4

Supplementary Figure S5

Supplementary Figure S6

Supplementary Method S1

Supplementary Table S1

Supplementary Table S2

Supplementary Table S3

Supplementary Data S1

Supplementary Data S2
