# Supplementary material for: VlbZIP30 of grapevine functions in dehydration tolerance via the abscisic acid core signaling pathway
Source: Hortic Res. 2018 Sep 1;5:49. doi: 10.1038/s41438-018-0054-x (PMC6119201; doi:10.1038/s41438-018-0054-x)
Supplement: Supplementary file 1 — Supplementary Figure S1 [file 41438_2018_54_MOESM1_ESM.pdf]

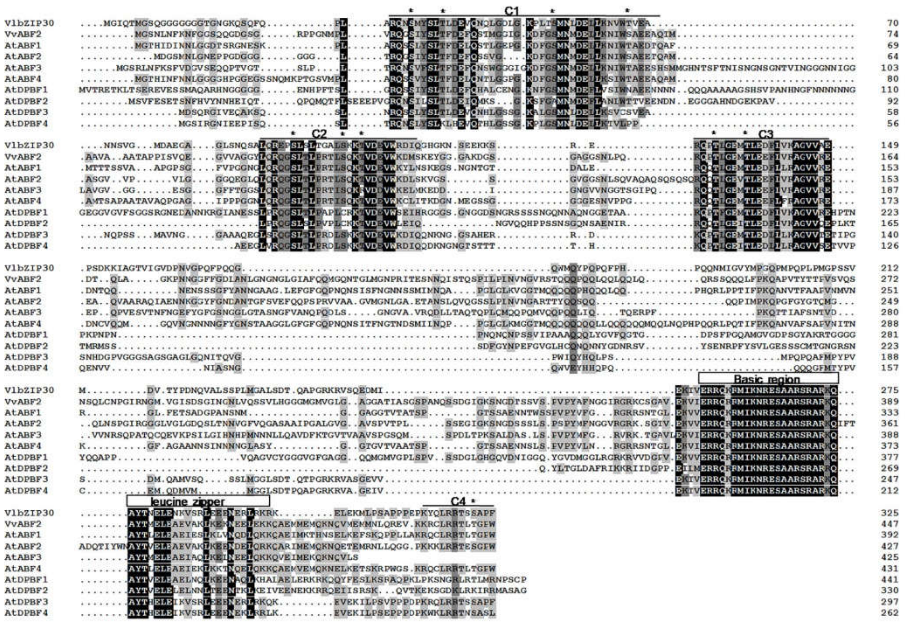

Figure S1. Multiple sequence alignment. Full-length sequence comparison of VlbZIP30 (VIT_13s0175g00120) with the ABF/DPBF subfamily of group A bZIP proteins from *Arabidopsis thaliana* (AT) and grapevine (*Vitis vinifera*, VIT). AtABF1 (AT1G49720), AtABF2 (AT1G45249), AtABF3 (AT4G34000), AtABF4 (AT3G19290), AtDPBF1 (AT2G36270), AtDPBF2 (AT3G44460), AtDPBF3 (AT3G56850), AtDPBF4 (AT2G41070) and VvABF2 (VIT_18s0001g10450) were used for the alignment. Conserved residues with 100%, 75-99 % or 33-75% amino identity are shaded in black, dark gray and light gray, respectively. The conserved bZIP domains are indicated with black rectangles. Putative phosphorylation sites (C1, C2, C3 and C4, underlined) are marked with asterisks.
