# Supplementary material for: VlbZIP30 of grapevine functions in dehydration tolerance via the abscisic acid core signaling pathway
Source: Hortic Res. 2018 Sep 1;5:49. doi: 10.1038/s41438-018-0054-x (PMC6119201; doi:10.1038/s41438-018-0054-x)
Supplement: Supplementary file 2 — Supplementary Figure S2 [file 41438_2018_54_MOESM2_ESM.pdf]

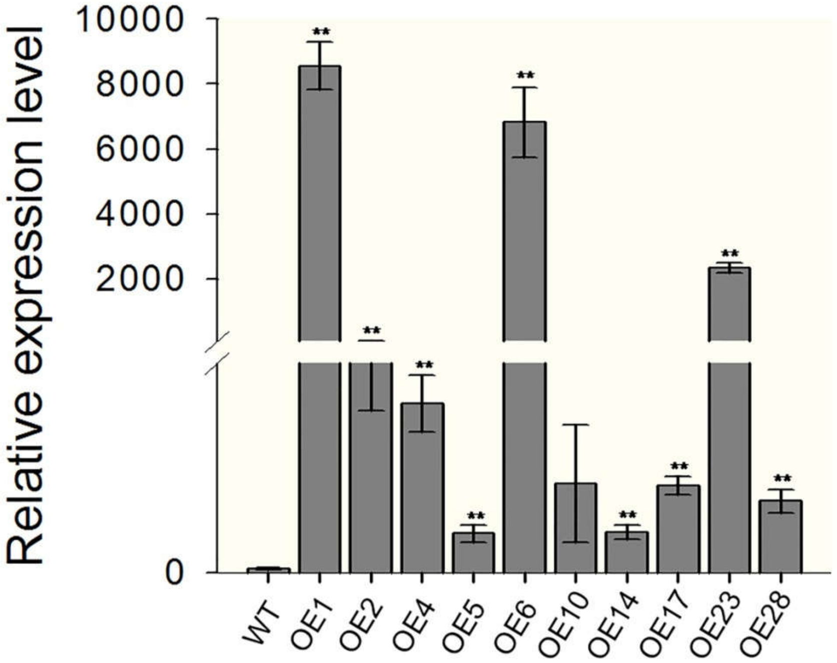


**Figure S2**. *VlbZIP30* mRNA levels in wild-type (WT) and transgenic plants analyzed by qRT-PCR. Data represent mean values ±SE from three independent experiments. Asterisks indicate statistical significance (**P < 0.01, Student's *t*-test) between the transgenic plants and WT plants.
