# Supplementary material for: VlbZIP30 of grapevine functions in dehydration tolerance via the abscisic acid core signaling pathway
Source: Hortic Res. 2018 Sep 1;5:49. doi: 10.1038/s41438-018-0054-x (PMC6119201; doi:10.1038/s41438-018-0054-x)
Supplement: Supplementary file 3 — Supplementary Figure S3 [file 41438_2018_54_MOESM3_ESM.pdf]

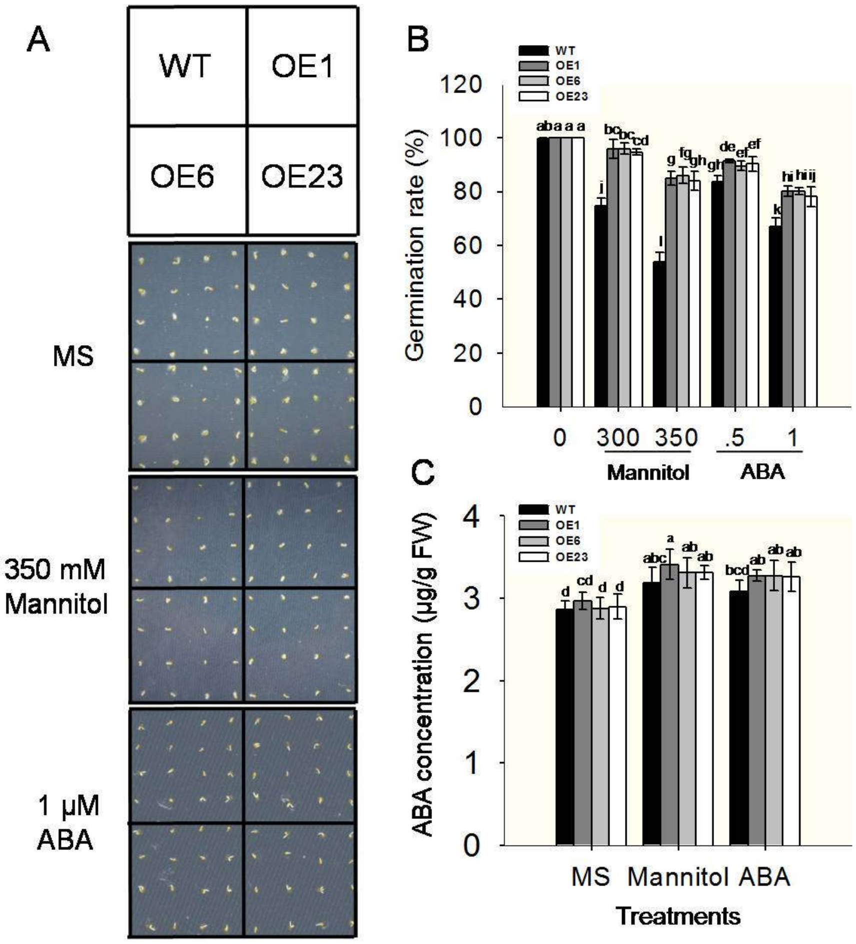

Figure S3. Phenotypes of wild type (WT) and *VlbZIP30* overexpressing transgenic lines at the seed germination stage under mannitol and abscisic acid (ABA) treatments. (A) Photographs of seed germination in WT and transgenic lines (OE1, OE6 and OE23) 3 d after seeds were cultivated on MS agar medium, with or without 350 mM mannitol or 1 μM ABA. (B) Seed germination rates of WT and transgenic lines 3 d after cultivation on Murashige-Skoog (MS) agar medium, or MS agar medium containing 300 or 350 mM mannitol or 0.5 or 1 μM ABA, respectively. (C) Endogenous ABA contents in WT and transgenic lines 3 d after cultivation on MS agar medium, or MS agar medium containing 350 mM mannitol or 1 μM ABA. Data represent mean values ±SE from three independent experiments. Statistically significant differences are indicated by different lowercase letters according to Fisher's LSD test ($P < 0.05$).
