# Supplementary material for: VlbZIP30 of grapevine functions in dehydration tolerance via the abscisic acid core signaling pathway
Source: Hortic Res. 2018 Sep 1;5:49. doi: 10.1038/s41438-018-0054-x (PMC6119201; doi:10.1038/s41438-018-0054-x)
Supplement: Supplementary file 4 — Supplementary Figure S4 [file 41438_2018_54_MOESM4_ESM.pdf]

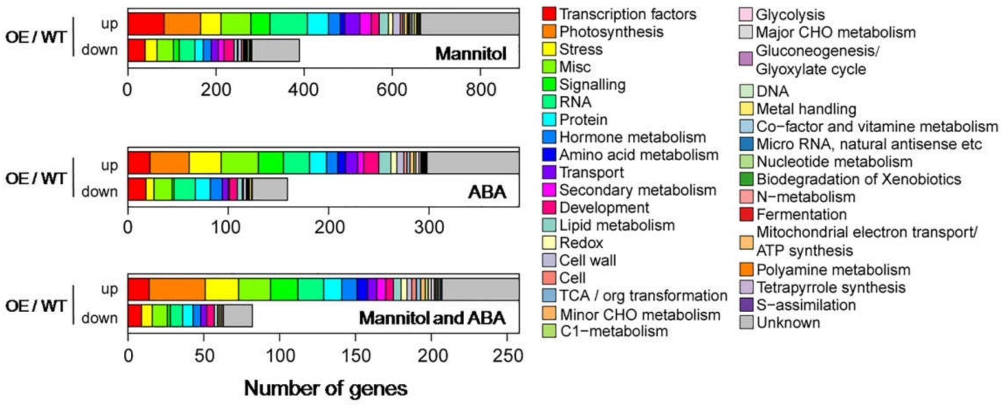

Figure S4. Gene ontology (GO) analyses of the differentially expressed genes (DEGs) in the *VlbZIP30*-overexpressing plants (OE) compared with wild type (WT) plants in response to abscisic acid (ABA) (OEA / WTA) or mannitol stress (OEM / WTM). Bar graphs separately display the numbers of DEGs classified into each GO category among the genes up- or down-regulated in the comparisons (OE / WT) induced by ABA, mannitol, or ABA and mannitol treatment.
