# Supplementary material for: VlbZIP30 of grapevine functions in dehydration tolerance via the abscisic acid core signaling pathway
Source: Hortic Res. 2018 Sep 1;5:49. doi: 10.1038/s41438-018-0054-x (PMC6119201; doi:10.1038/s41438-018-0054-x)
Supplement: Supplementary file 5 — Supplementary Figure S5 [file 41438_2018_54_MOESM5_ESM.pdf]

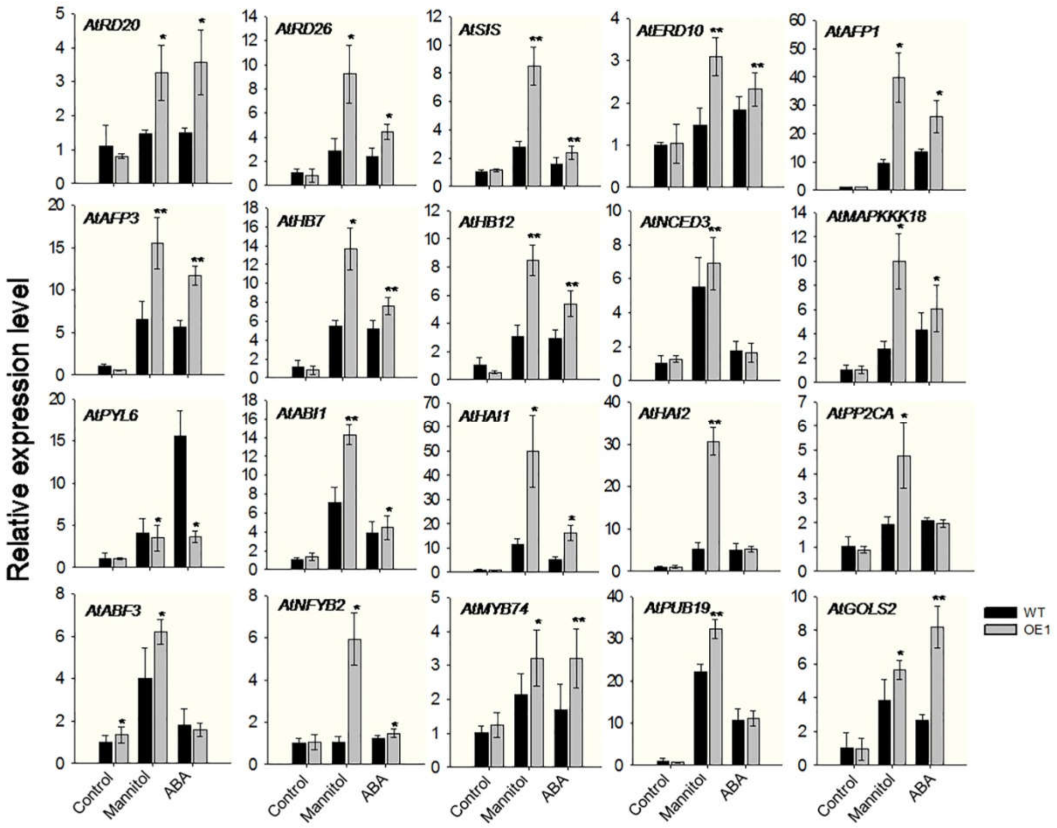

Figure S5. Experimental validation of transcriptome data by expression analysis of representative drought-responsive genes in *Arabidopsis thaliana* using qRT-PCR. The expression level in the wild type (WT) plants under control conditions was defined as 1.0. The *AtActin2* gene was used as an internal control. Data represent mean values ±SE from three independent experiments. Asterisks indicate statistical significance (*0.01 < P < 0.05, **P < 0.01, Student's *t*-test) between the overexpressing (OE) lines and WT plants.
