# Supplementary material for: VlbZIP30 of grapevine functions in dehydration tolerance via the abscisic acid core signaling pathway
Source: Hortic Res. 2018 Sep 1;5:49. doi: 10.1038/s41438-018-0054-x (PMC6119201; doi:10.1038/s41438-018-0054-x)
Supplement: Supplementary file 6 — Supplementary Figure S6 [file 41438_2018_54_MOESM6_ESM.pdf]

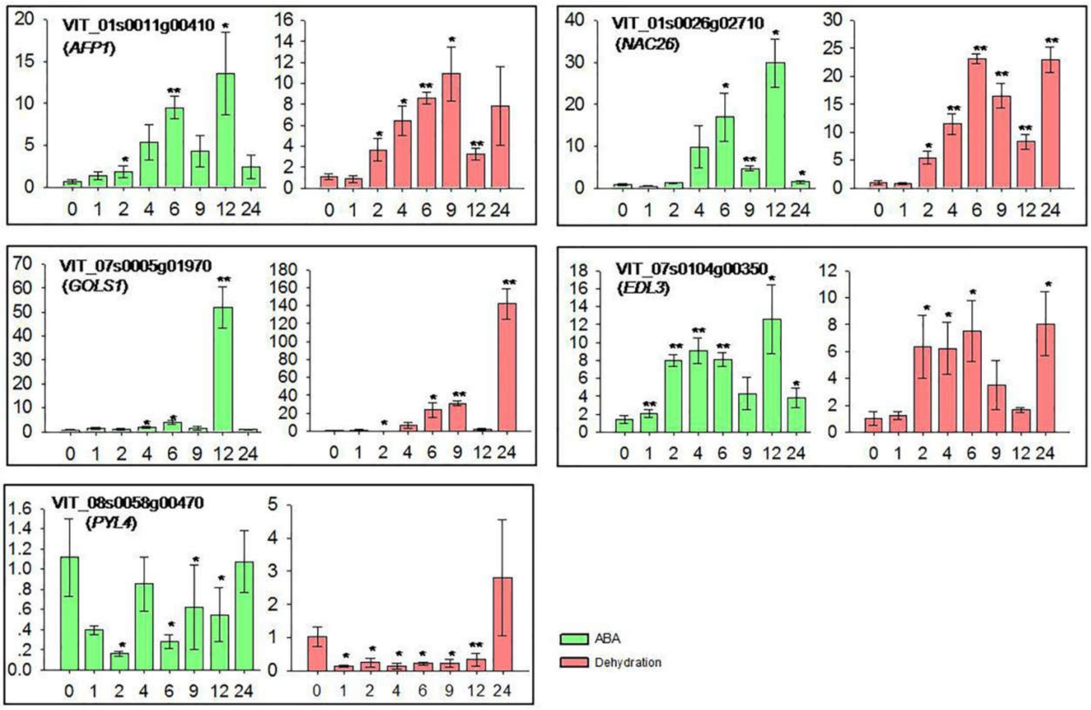

Figure S6. Gene expression profiles of selected *VlbZIP30*-induced grapevine candidate genes analyzed using qRT-PCR. For each gene, the expression level at 0 h for the abscisic acid (ABA) and dehydration treatments was defined as 1.0. The *VvACTIN1* gene was used as an internal control. Data represent mean values ±SE from three independent experiments. Asterisks indicate statistical significance (*0.01 < P < 0.05, **P < 0.01, Student's *t*-test) between the treated and untreated control plants.
