# Supplementary material for: VlbZIP30 of grapevine functions in dehydration tolerance via the abscisic acid core signaling pathway
Source: Hortic Res. 2018 Sep 1;5:49. doi: 10.1038/s41438-018-0054-x (PMC6119201; doi:10.1038/s41438-018-0054-x)
Supplement: Supplementary file 7 — Supplementary Method S1 [file 41438_2018_54_MOESM7_ESM.pdf]

Vector construction

Total RNA was extracted from the grape leaves as previously described.[1] First-strand cDNA was synthesized using PrimerScript^{TM}Reverse Transcriptase (TaKaRa Biotechnology), according to the manufacturer's instructions. To obtain a 35S:*VlbZIP30* vector, the open reading frame (ORF) of *VlbZIP30* was amplified by PCR using the specific primers F1 (5′-CGC TCT AGA ATG GGG ATT CAG ACT ATG GGG-3′ *Xba*I site underlined) and R1 (5′-GGC GGT ACC TCA GAA TGG GGC TGA ACT C-3′ *Kpn*I site underlined). The PCR product was cloned into the pGEM-T Easy vector (Promega, Madison, WI, USA), and the resulting plasmid (pGEM-Teasy-*VlbZIP30*) was sequenced to confirm sequence fidelity. Then the ORF of *VlbZIP30* (with *Xba*I and *Kpn*I sites added to the 5′ and 3′ end, respectively) was amplified from the pGEM-Teasy-*VlbZIP30* vector, and inserted immediately downstream of the CaMV 35S promoter in the plant overexpression vector, pCambia2300 (Cambia, Brisbane, QLD, Australia).

DNA was extracted from the grape leaves as previously described [1]. To obtain a $\text{Pro}_{\textit{VlbZIP30}}$:*GUS* (β-glucuronidase) vector, a 2,000 bp fragment upstream of the *VlbZIP30* translation start site was amplified by PCR from the genomic DNA using the specific primers F2 (5′-GAC CAT GAT TAC GCC AAG CTT TTC CCC AAC CCT ACC CTA C-3′ *HindIII* site underline) and R2 (5′-ACC ACC CGG GGA TCC TCT AGA TCA ACA ACC CCC ACC TAA TT-3′ *XbaI* site underlined), re-sequenced and inserted into the binary vector pBI121 (Clontech Lab. Inc., Palo Alto, CA, USA) containing the *GUS* reporter gene. The integrity of the final constructs was confirmed by sequencing.

References

1. Tu, M. X., Wang, X. H., Feng, T. Y., et al. 2016, Expression of a grape (Vitis vinifera) bZIP transcription factor, VIbZIP36, in Arabidopsis thaliana confers tolerance of drought stress during seed germination and seedling establishment. *Plant Sci*, **252**, 311-323.
