# Supplementary material for: VlbZIP30 of grapevine functions in dehydration tolerance via the abscisic acid core signaling pathway
Source: Hortic Res. 2018 Sep 1;5:49. doi: 10.1038/s41438-018-0054-x (PMC6119201; doi:10.1038/s41438-018-0054-x)
Supplement: Supplementary file 8 — Supplementary Table S1 [file 41438_2018_54_MOESM8_ESM.pdf]

Table S1. Specific primers used for qRT-PCR. F, forward; R, reverse.

Gene ID	Gene names	Primer sequences (5’-3’)
AT2G33380	*AtRD20*	F: TACACTTCCGAGTTGGGTGC
		R: AACCGTTAGCGCGTATTTGC
AT4G27410	*AtRD26*	F: GGGTGTTAGAGAGAAAGATCCGTTA
		R: TGTTTGCTTACTTGGCAAATCC
AT5G02020	*AtSIS*	F: GACTGTGACTGGTGGTTGCT
		R: TTCTTGTTCGTAGAGTTGCTGG
AT1G20450	*AtERD10*	F: ATCGGCGCCAGAGATTAAGG
		R: AAGAGCTGTTGGATCGGTGG
AT1G69260	*AtAFP1*	F: ATGGAGCCACAAGGGAAAGG
		R: GAGGACCACCGGCATGTTTA
AT3G29575	*AtAFP3*	F: GGCAAAGAAGCGAGCCAAAA
		R: ACCACCAGCATGCTTAACGA
AT2G46680	*AtHB7*	F: AGCGACGAGCAGATCAAGTC
		R: GCTTGGATTTCCAACGAGCC
AT3G61890	*AtHB12*	F: CTGCAACCAAGACAAGTGGC
		R: GCCAGTCCTTGATCACCACA
AT3G14440	*AtNCED3*	F: TTGATGCTCCAGATTGCTTC
		R: GGACCCTATCACGACGACTT
AT1G05100	*AtMAPKKK18*	F: TTACCAGAGCTGCCTTGCTC
		R: GCTTGAACGGTCCTCTGACA
AT4G34000	*AtABF3*	F: CTTTGTTGATGGTGTGAGTGAG
		R: GTGTTTCCACTATTACCATTGC
AT4G26080	*AtABI1*	F: CGGTTCTCAGGTAGCGAAC
		R: TCACCATCGCAGAGCATC
AT5G59220	*AtHAI1*	F: CCATGGCTGTTCCCATGTA
		R: AAGCTACGCGCCATTGAC
AT1G07430	*AtHAI2*	F: GGTGCAAGAGTCTTAGGCGT
		R: TCTTCCTCAGTCCGATCCGT
AT3G11410	*AtPP2CA*	F: GGCGAGTGATGGACTATGGG
		R: TTTGTTAAGAGCAACGCCGC
AT2G40330	*AtPYL6*	F: GAGCGGCTTGAGATCATGGA
		R: ACACGTGTCCTCTTCTTGCC

AT4G05100	*AtMYB74*	F: CCAATGAAGACGACGGTCCA
		R: TGAGTTTAACGGAGTCGGGC
AT5G47640	*AtNFYB2*	F: GAGGGCAAAACGGGAACAAC
		R: AGCCCAGAGCAAATCGTCTC
AT1G56600	*AtGolS2*	F: CGCCACAGTACAAGATCGGT
		R: AGTGGAGAGGTTAGGCTCGT
AT1G60190	*AtPUB19*	F: TCGACACGTTTCCAGTCAGG
		R: TAGCTCGTTTGTCGTCTCGG
AT1G68530	*AtKCS6*	F: GTGGCGAGCCAAATACAAGC
		R: AGGACCAAAGGACCTATTGTGG
AT1G64400	*AtLACS3*	F: TATACAGTCATGTCGCCGCC
		R: TTTGGCCCAATGCTCGATCT
AT5G62470	*AtMYB96*	F: CAAGCTCTTTGTGAGGCCTTG
		R: ACAACGGTCAAGCAAAGCTG
AT3G18780	*AtActin2*	F: AGTGTCTGGATCGGTGGTTC
		R: CCCCAGCTTTTTAAGCCTTT
VIT_13S0175G00120	*VlbZIP30*	F: GGGGATTCAGACTATGGG
		R: ATTAGCCTCAACCGTCCA
VIT_00S0203G00070	--	F: TCAGCGATTGGCAAAGCAAC
		R: GCTTCCGTAGCTGATCTCCC
VIT_12S0059G01640	--	F: CGATCACTCCGAGGAAGACG
		R: TCCGACACTTGACTTCCAGC
VIT_14S0108G00450	--	F: GGAATCGCGAGGTGGAATCT
		R: CGACTCGAACTCCTCCGATG
VIT_05S0020G03530	*VvLEA-D34*	F: CAGTCCGACCAAGAGCCAAT
		R: AAGACCAGCTCTCTCGTTGC
VIT_01S0011G00410	*VvAFP1*	F: GGGAACAGTGGAGAGCAGTC
		R: TACGAAACAGCGGGAGTCAC
VIT_01S0026G02710	*VvNAC26*	F: AATTGCCTGAGAAGGCGGAA
		R: GTAGCCGGACACTGTAGCTC
VIT_06S0004G05460	*VvPP2C9*	F: CGATCCCTCTCTCCTCCGAT
		R: CCCAAGCCTTCCCCTTCAG
VIT_06S0004G06830	--	F: CCCGGGTCCCCTTTTTAGAC
		R: CCAACTGTAGCCGACACCAT

VIT_07S0005G01970	*VvGolS1*	F: CTTCGGGAAAACCGTCCAGA
		R: TCTGGTTCTCGGGTGGGTAA
VIT_07S0104G00350	*VvEDL3*	F: AAGACGAGGGCGTGTTTGAT
		R: ACACAAAGTACTCCAGCCCG
VIT_08S0007G01360	--	F: TCCGTCTTCCACGGGTATCT
		R: TAGTCCCTGGTTTGGCGTTC
VIT_13S0019G02200	*VvPP2C37*	F: ATTTGTTGCCGGTGTTTCCG
		R: GGCATTCCTGGACCAACTGA
VIT_14S0171G00360	--	F: AGGATGGTGAGCCAGGAGAT
		R: AGCGACCTAAACTCAACGCA
VIT_17S0000G08080	*VvPUB19*	F: AGGTTAGAGTCCCGGCAGAT
		R: AGCACTCTGGGGCAATTCTC
VIT_18S0001G10450	*VvABF2*	F: CCCTTGGTGAGACAAGGGTC
		R: TACCGCAGCCATAGTTTGGG
VIT_19S0014G03290	*VvNAC17*	F: TGAGAGTTTCACAGCCGGAC
		R: TACTGGGTCGACTCTCTGGG
VIT_08S0058G00470	*VvPYL4*	F: ATCCACAGGCCTACAAGCAC
		R: CTTTGGTGTTTCCTGGGGGT
VIT_02S0025G01410	*VvLACS4*	F: CCTTCGCCAGGGATGGATTT
		R: CCAAGCATGCGATTTCCAGG
VIT_14S0006G02990	*VvKCS6*	F: CTTGAGAGGTCTGGCCTTGG
		R: GAAGGGGTGGGGGAAAAGAG
VIT_04S0044G00580	*VvActin1*	F: GATTCTGGTGATGGTGTGAGT
		R: GACAATTTCCCGTTCAGCAGT
