# Supplementary material for: VlbZIP30 of grapevine functions in dehydration tolerance via the abscisic acid core signaling pathway
Source: Hortic Res. 2018 Sep 1;5:49. doi: 10.1038/s41438-018-0054-x (PMC6119201; doi:10.1038/s41438-018-0054-x)
Supplement: Supplementary file 9 — Supplementary Table S2 [file 41438_2018_54_MOESM9_ESM.pdf]

Table S2. The differentially expressed genes in the *VlbZIP30* transgenic plants (OE / WT) with expression changes (FDR<0.05) of at least twofold under control condition from the transcriptome data.

		OEC / WTC		OEM / WTM		OEA / WTA	
**AGI**	**Symbol**	**Log_2FC**	**FDR**	**Log_2FC**	**FDR**	**Log_2FC**	**FDR**
Up-regulated genes							
AT1G27565	--	3.04	1.26E-24	2.52	8.41E-35	1.97	3.22E-14
AT5G06630	--	1.55	0.027	-0.99	2.32E-08	-0.24	0.6484
AT4G25790	--	1.48	0.011	-0.47	0.118	-0.02	0.9729
AT2G24980	--	1.47	0.023	-0.98	4.75E-06	-0.23	0.6368
AT5G50240	PIMT2	1.20	0.038	0.17	0.478	0.13	0.7569
AT3G29410	TPS25	1.09	6.06E-07	0.10	0.647	-0.10	0.7522
AT5G50560	--	1.06	0.041	0.60	0.014	0.582	0.2322
AT1G48930	GH9C1	1.04	0.013	-0.61	0.0004	0.14	0.6695
AT5G49870	--	2.62	0.0003			2.88	1.91E-07
AT5G49770	--	2.24	5.82E-05				
Down-regulated genes							
AT5G56380	--	-10.81	3.44E-62	-6.83	1.72E-120	-6.77	4.47E-103
AT5G56370	--	-8.46	3.73E-17	-8.21	2.73E-22	-8.06	1.01E-18
AT5G54030	--	-3.88	4.36E-13	-4.23	8.15E-22	-3.62	1.01E-21
AT3G47965	--	-3.88	5.91E-09	-3.02	2.68E-10	-3.38	2.64E-10
newGene505	--	-2.78	4.92E-46	-3.42	1.54E-137	-3.07	3.54E-81
AT5G56910	--	-1.19	0.0042	-0.49	0.0145	-0.82	0.0008
AT5G54520	--	-1.07	0.0486	-0.86	0.0003	-1.07	7.77E-05
AT5G53750	--	-1.01	0.0228	-0.87	0.0002	-0.62	0.0249
AT5G20790	--	-1.01	0.0323	0.55	0.1983	1.58	6.02E-05
AT5G56840	--	-2.15	0.0009				

<-4 -4~-2 -2~0 0~1 1~2 2~3 >3

FDR, false discovery rate

FC, fold change

OE / WT, overexpression line / wild type

C, control

M, mannitol

A, ABA
