# Supplementary material for: VlbZIP30 of grapevine functions in dehydration tolerance via the abscisic acid core signaling pathway
Source: Hortic Res. 2018 Sep 1;5:49. doi: 10.1038/s41438-018-0054-x (PMC6119201; doi:10.1038/s41438-018-0054-x)
Supplement: Supplementary file 10 — Supplementary Table S3 [file 41438_2018_54_MOESM10_ESM.pdf]

Table S3. Selected genes involved in other biological processes based on expression level differences (FDR<0.05) of at least two-fold in the *VlbZIP30* transgenic plants under ABA or mannitol stress treatment from the transcriptome data.

Locus	Symbol	Description	Reference	Log$_2$FC (OE / WT) C	M	A
**Wax biosynthesis**						
AT2G28630	KCS12*	Ketoacy-CoA synthase	1	-0.189	2.141	-0.196
AT1G68530	KCS6*	Ketoacy-CoA synthase	1	-0.003	1.456	0.474
AT1G64400	LACS3*	Long-chain acyl-CoA synthetase	1	-0.237	1.433	0.973
AT3G55130	ABCG19	ABC transporter	1	-0.033	1.230	0.805
AT5G59320	LTP3*	Nonspecific lipid-transfer protein	1	-0.144	0.285	-1.205
**Photosynthesis**						
AT2G05100	LHCB2.1	Light-harvesting chlorophyll A/B binding protein	2	-0.311	1.240	0.651
AT2G05070	LHCB2.2	Light-harvesting chlorophyll A/B binding protein	2	-0.294	1.601	0.818
AT3G27690	LHCB2.4	Light-harvesting chlorophyll A/B binding protein	3	-0.103	1.920	1.153
AT5G54270	LHCB3	Light-harvesting chlorophyll A/B binding protein	4	-0.059	1.417	0.809
AT3G47470	LHCA4	Light-harvesting chlorophyll A/B binding protein	5	-0.040	1.381	0.995
AT3G08940	LHCB4.2	Light-harvesting chlorophyll A/B binding protein	6	-0.168	1.438	1.031
**Brassinosteroid signaling**						
AT1G73830	BEE3	bHLH transcription factor	7	-0.020	-0.195	-1.563
AT3G57130	BSS1	Brz-sensitive-short hypocotyl	8	-0.262	-1.081	-0.091
AT4G08950	EXO	phosphate-responsive family protein	9	0.153	-1.184	-0.367
**Ethylene signaling**						
AT1G04310	ERS2	ethylene response sensor 2	10	0.132	-0.027	-1.107
AT3G23150	ETR2	ethylene response 2	10	0.125	-0.484	-1.422
AT5G25350	EBF2	EIN3-binding F-box protein 2	11	0.122	-0.481	-1.600
AT3G59900	ARGOS	auxin-regulated gene involved in organ size	12	0.114	-1.302	-0.866
**Cytokinins signaling**						
AT1G67110	CYP735A2	cytochrome P450	13	0.129	-0.326	-1.772
**Fe homeostasis**						
AT4G19690	IRT1	Iron-regulated transporter	14	0.669	3.394	0.438
AT3G56970	BHLH038	bHLH transcription factor	15	-0.195	4.434	1.021
AT3G56980	BHLH039	bHLH transcription factor	15	0.123	2.793	1.035
AT2G41240	BHLH100	bHLH transcription factor	15	-0.117	3.110	1.908
AT5G04150	BHLH101	bHLH transcription factor	15	0.020	ND	1.742
**Ca^{2+} homeostasis**						
AT1G03445	BSU1	Calcineurin-like phosphoesterase	16	ND	1.573	1.053
AT5G10930	CIPK5	Ca^{2+} B-like interacting protein kinases	17	0.145	1.444	0.300
AT5G45810	CIPK19	Ca^{2+} B-like interacting protein kinases	18	ND	-0.283	1.236
**Phosphate starvation-related gene**						
AT1G73010	PS2	induced by phosphate starvation	19	-0.939	0.284	1.487
AT5G20150	SPX1	induced by phosphate starvation	20	-0.566	0.527	1.120
AT5G20410	MGD2	induced by phosphate starvation	21	-0.666	0.615	1.191

AT2G45130	SPX3	induced by phosphate starvation [22]	-0.734	ND	1.745
**Sulfur deficiency-related gene**					
AT2G44460	BGLU28	sulfur deficiency-activated gene [23]	-0.246	ND	2.526
AT3G49580	LSU1	response to low sulfur [24]	-0.419	-0.060	1.426
AT5G24660	LSU2	response to low sulfur [24]	-0.612	-0.430	1.704
AT5G48850	ATSDI1	sulfur deficiency-induced gene [25]	-0.699	-1.157	1.537
**Disease**					
AT5G24770	VSP2	Vegetative storage protein 2 [26]	ND	1.489	0.499
AT3G28007	SWEET4	Nodulin MtN3 family protein [27]	ND	1.580	ND
AT1G44830	ERF014	ethylene response factor [28]	0.349	-1.191	-0.259
AT2G26440	PME12	pectin methylesterases [29]	-0.115	-1.138	-0.986
AT2G30750	CYP71A12	pathogen-induced P450 [30]	-1.127	-0.945	-2.467
**Transporter**					
AT5G03490	UGT89A2	UDP-glucosyl transferase [31]	-0.752	1.225	0.631
AT3G50760	GATL2	Galacturonosyl transferase [32]	0.483	1.749	0.425
**Enzyme**					
AT1G62540	$FMO_{GS\text{-}OX2}$	flavin-monooxygenases enzyme [33]	-0.312	2.441	1.071
AT3G47340	ASN1	asparagines synthase [34]	ND	-0.535	1.237
AT4G15440	HPL1	Hydroperoxide lyase 1 [35]	-0.108	1.736	1.258

<-1   -1~0   0~1   1~2   >2   ND

FDR, false discovery rate

FC, fold change

OE / WT, overexpression line / wild type

M, mannitol

A, ABA

ND, not detected.

*, not included in the selected genes

References

1. Seo, P. J., Lee, S. B., Suh, M. C., Park, M. J., Go, Y. S. and Park, C. M. 2011, The MYB96 transcription factor regulates cuticular wax biosynthesis under drought conditions in Arabidopsis. *Plant Cell*, **23**, 1138-1152.
2. Mishra, Y., Jankanpaa, H. J., Kiss, A. Z., Funk, C., Schroder, W. P. and Jansson, S. 2012, Arabidopsis plants grown in the field and climate chambers significantly differ in leaf morphology and photosystem components. *BMC plant biology*, **12**, 6.
3. Shaikhali, J., Noren, L., de Dios Barajas-Lopez, J., et al. 2012, Redox-mediated mechanisms regulate DNA binding activity of the G-group of basic region leucine zipper (bZIP) transcription factors in Arabidopsis. *The Journal of biological chemistry*, **287**, 27510-27525.
4. Pietrzykowska, M., Suorsa, M., Semchonok, D. A., et al. 2014, The light-harvesting chlorophyll a/b binding proteins Lhcb1 and Lhcb2 play complementary roles during state transitions in Arabidopsis. *Plant Cell*, **26**, 3646-3660.
5. Wientjes, E., Roest, G. and Croce, R. 2012, From red to blue to far-red in Lhca4: how does the protein modulate the spectral properties of the pigments? *Biochimica et biophysica acta*, **1817**,

711-717.

6. de Bianchi, S., Betterle, N., Kouril, R., et al. 2011, Arabidopsis mutants deleted in the light-harvesting protein Lhcb4 have a disrupted photosystem II macrostructure and are defective in photoprotection. *Plant Cell*, **23**, 2659-2679.
7. Friedrichsen, D. M., Nemhauser, J., Muramitsu, T., et al. 2002, Three redundant brassinosteroid early response genes encode putative bHLH transcription factors required for normal growth. *Genetics*, **162**, 1445-1456.
8. Shimada, S., Komatsu, T., Yamagami, A., et al. 2015, Formation and dissociation of the BSS1 protein complex regulates plant development via brassinosteroid signaling. *Plant Cell*, **27**, 375-390.
9. Schroder, F., Lisso, J., Lange, P. and Mussig, C. 2009, The extracellular EXO protein mediates cell expansion in Arabidopsis leaves. *BMC plant biology*, **9**, 20.
10. Ge, X. M., Cai, H. L., Lei, X., Zhou, X., Yue, M. and He, J. M. 2015, Heterotrimeric G protein mediates ethylene-induced stomatal closure via hydrogen peroxide synthesis in Arabidopsis. *The Plant journal : for cell and molecular biology*, **82**, 138-150.
11. Li, W., Ma, M., Feng, Y., et al. 2015, EIN2-directed translational regulation of ethylene signaling in Arabidopsis. *Cell*, **163**, 670-683.
12. Rai, M. I., Wang, X., Thibault, D. M., et al. 2015, The ARGOS gene family functions in a negative feedback loop to desensitize plants to ethylene. *BMC plant biology*, **15**, 157.
13. Kiba, T., Takei, K., Kojima, M. and Sakakibara, H. 2013, Side-chain modification of cytokinins controls shoot growth in Arabidopsis. *Developmental cell*, **27**, 452-461.
14. Leskova, A. and Giehl, R. F. H. 2017, Heavy Metals Induce Iron Deficiency Responses at Different Hierarchic and Regulatory Levels. **174**, 1648-1668.
15. Van Dingenen, J., Antoniou, C., Filippou, P. and Pollier, J. 2017, Strobilurins as growth-promoting compounds: how Stroby regulates Arabidopsis leaf growth. **40**, 1748-1760.
16. Kim, T. W., Guan, S., Burlingame, A. L. and Wang, Z. Y. 2011, The CDG1 kinase mediates brassinosteroid signal transduction from BRI1 receptor kinase to BSU1 phosphatase and GSK3-like kinase BIN2. *Molecular cell*, **43**, 561-571.
17. Schlucking, K., Edel, K. H., Koster, P., et al. 2013, A new beta-estradiol-inducible vector set that facilitates easy construction and efficient expression of transgenes reveals CBL3-dependent cytoplasm to tonoplast translocation of CIPK5. *Molecular plant*, **6**, 1814-1829.
18. Zhou, L., Lan, W., Chen, B., Fang, W. and Luan, S. 2015, A calcium sensor-regulated protein kinase, CALCINEURIN B-LIKE PROTEIN-INTERACTING PROTEIN KINASE19, is required for pollen tube growth and polarity. *Plant Physiol*, **167**, 1351-1360.
19. Chandrika, N. N., Sundaravelpandian, K., Yu, S. M. and Schmidt, W. 2013, ALFIN-LIKE 6 is involved in root hair elongation during phosphate deficiency in Arabidopsis. *The New phytologist*, **198**, 709-720.
20. Zhao, L., Liu, F., Xu, W., et al. 2009, Increased expression of OsSPX1 enhances cold/subfreezing tolerance in tobacco and Arabidopsis thaliana. *Plant biotechnology journal*, **7**, 550-561.
21. Kobayashi, K., Narise, T., Sonoike, K., et al. 2013, Role of galactolipid biosynthesis in coordinated development of photosynthetic complexes and thylakoid membranes during chloroplast biogenesis in Arabidopsis. *The Plant journal : for cell and molecular biology*, **73**, 250-261.
22. Franco-Zorrilla, J. M., Martin, A. C., Leyva, A. and Paz-Ares, J. 2005, Interaction between phosphate-starvation, sugar, and cytokinin signaling in Arabidopsis and the roles of cytokinin receptors CRE1/AHK4 and AHK3. *Plant Physiol*, **138**, 847-857.

23. Zhang, B., Pasini, R., Dan, H., et al. 2014, Aberrant gene expression in the Arabidopsis SULTR1;2 mutants suggests a possible regulatory role for this sulfate transporter in response to sulfur nutrient status. *The Plant journal : for cell and molecular biology*, **77**, 185-197.
24. Lewandowska, M., Wawrzynska, A., Moniuszko, G., et al. 2010, A contribution to identification of novel regulators of plant response to sulfur deficiency: characteristics of a tobacco gene UP9C, its protein product and the effects of UP9C silencing. *Molecular plant*, **3**, 347-360.
25. Howarth, J. R., Parmar, S., Barraclough, P. B. and Hawkesford, M. J. 2009, A sulphur deficiency-induced gene, sdi1, involved in the utilization of stored sulphate pools under sulphur-limiting conditions has potential as a diagnostic indicator of sulphur nutritional status. *Plant biotechnology journal*, **7**, 200-209.
26. Vadassery, J., Reichelt, M., Hause, B., Gershenzon, J., Boland, W. and Mithofer, A. 2012, CML42-mediated calcium signaling coordinates responses to Spodoptera herbivory and abiotic stresses in Arabidopsis. *Plant Physiol*, **159**, 1159-1175.
27. Chong, J., Piron, M. C., Meyer, S., Merdinoglu, D., Bertsch, C. and Mestre, P. 2014, The SWEET family of sugar transporters in grapevine: VvSWEET4 is involved in the interaction with Botrytis cinerea. *J Exp Bot*, **65**, 6589-6601.
28. Zhang, H., Hong, Y., Huang, L., Li, D. and Song, F. 2016, Arabidopsis AtERF014 acts as a dual regulator that differentially modulates immunity against Pseudomonas syringae pv. tomato and Botrytis cinerea. *Scientific reports*, **6**, 30251.
29. Bethke, G., Grundman, R. E., Sreekanta, S., Truman, W., Katagiri, F. and Glazebrook, J. 2014, Arabidopsis PECTIN METHYLESTERASEs contribute to immunity against Pseudomonas syringae. *Plant Physiol*, **164**, 1093-1107.
30. Rajniak, J., Barco, B., Clay, N. K. and Sattely, E. S. 2015, A new cyanogenic metabolite in Arabidopsis required for inducible pathogen defence. *Nature*, **525**, 376-379.
31. Chen, H. Y. and Li, X. 2017, Identification of a residue responsible for UDP-sugar donor selectivity of a dihydroxybenzoic acid glycosyltransferase from Arabidopsis natural accessions. *The Plant journal : for cell and molecular biology*, **89**, 195-203.
32. Lao, N. T., Long, D., Kiang, S., et al. 2003, Mutation of a family 8 glycosyltransferase gene alters cell wall carbohydrate composition and causes a humidity-sensitive semi-sterile dwarf phenotype in Arabidopsis. *Plant Mol Biol*, **53**, 647-661.
33. Li, J., Kristiansen, K. A., Hansen, B. G. and Halkier, B. A. 2011, Cellular and subcellular localization of flavin-monooxygenases involved in glucosinolate biosynthesis. *J Exp Bot*, **62**, 1337-1346.
34. Hanson, J., Hanssen, M., Wiese, A., Hendriks, M. M. and Smeekens, S. 2008, The sucrose regulated transcription factor bZIP11 affects amino acid metabolism by regulating the expression of ASPARAGINE SYNTHETASE1 and PROLINE DEHYDROGENASE2. *The Plant journal : for cell and molecular biology*, **53**, 935-949.
35. Nilsson, A. K., Fahlberg, P., Johansson, O. N., Hamberg, M., Andersson, M. X. and Ellerstrom, M. 2016, The activity of HYDROPEROXIDE LYASE 1 regulates accumulation of galactolipids containing 12-oxo-phytodienoic acid in Arabidopsis. *J Exp Bot*, **67**, 5133-5144.
